# Phytochemicals in the treatment of inflammation-associated diseases: the journey from preclinical trials to clinical practice

**DOI:** 10.3389/fphar.2023.1177050

**Published:** 2023-05-09

**Authors:** Akib Nisar, Suresh Jagtap, Suresh Vyavahare, Manasi Deshpande, Abhay Harsulkar, Prabhakar Ranjekar, Om Prakash

**Affiliations:** ^1^ Biochemical Sciences Division, Rajiv Gandhi Institute of IT and Biotechnology, Bharati Vidyapeeth Deemed to be University, Pune, Maharashtra, India; ^2^ Herbal Medicine, Interactive Research School for Health Affairs, Bharati Vidyapeeth Deemed to be University, Pune, Maharashtra, India; ^3^ Shatayu Ayurved and Research Centre, Solapur, Maharashtra, India; ^4^ Department of Dravyagun Vigyan, College of Ayurved, Bharati Vidyapeeth Deemed to be University, Pune, Maharashtra, India; ^5^ Pharmaceutical Biotechnology, Poona College of Pharmacy, Bharati Vidyapeeth Deemed to be University, Pune, Maharashtra, India; ^6^ Innovation Biologicals Pvt., Ltd., Pune, Maharashtra, India; ^7^ Department of Microbiology, Immunology and Parasitology, University Health Sciences Center, New Orleans, LA, United States; ^8^ Stanley S. Scott Cancer Center, Louisiana State University Health Sciences Center, New Orleans, LA, United States

**Keywords:** inflammation, phytochemical, medicinal drug, preclinical, clinical

## Abstract

Advances in biomedical research have demonstrated that inflammation and its related diseases are the greatest threat to public health. Inflammatory action is the pathological response of the body towards the external stimuli such as infections, environmental factors, and autoimmune conditions to reduce tissue damage and improve patient comfort. However, when detrimental signal-transduction pathways are activated and inflammatory mediators are released over an extended period of time, the inflammatory process continues and a mild but persistent pro-inflammatory state may develop. Numerous degenerative disorders and chronic health issues including arthritis, diabetes, obesity, cancer, and cardiovascular diseases, among others, are associated with the emergence of a low-grade inflammatory state. Though, anti-inflammatory steroidal, as well as non-steroidal drugs, are extensively used against different inflammatory conditions, they show undesirable side effects upon long-term exposure, at times, leading to life-threatening consequences. Thus, drugs targeting chronic inflammation need to be developed to achieve better therapeutic management without or with a fewer side effects. Plants have been well known for their medicinal use for thousands of years due to their pharmacologically active phytochemicals belonging to diverse chemical classes with a number of these demonstrating potent anti-inflammatory activity. Some typical examples include colchicine (alkaloid), escin (triterpenoid saponin), capsaicin (methoxy phenol), bicyclol (lignan), borneol (monoterpene), and quercetin (flavonoid). These phytochemicals often act via regulating molecular mechanisms that synergize the anti-inflammatory pathways such as increased production of anti-inflammatory cytokines or interfere with the inflammatory pathways such as to reduce the production of pro-inflammatory cytokines and other modulators to improve the underlying pathological condition. This review describes the anti-inflammatory properties of a number of biologically active compounds derived from medicinal plants, and their mechanisms of pharmacological intervention to alleviate inflammation-associated diseases. The emphasis is given to information on anti-inflammatory phytochemicals that have been evaluated at the preclinical and clinical levels. Recent trends and gaps in the development of phytochemical-based anti-inflammatory drugs have also been included.

## 1 Introduction

**FIGURE 1:**
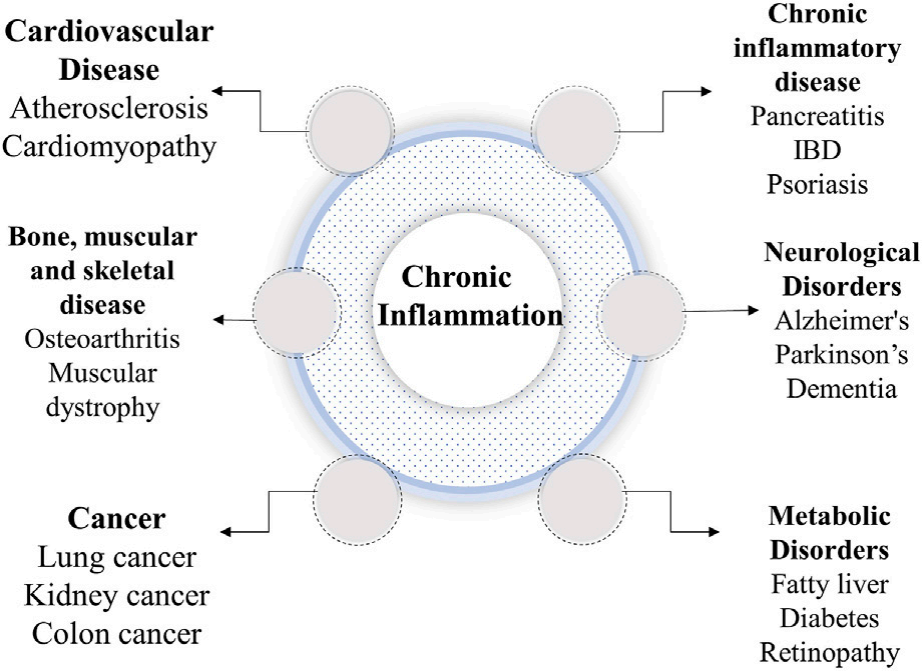
Complications caused by chronic inflammation.

Chronic inflammation and associated disorders are the biggest public health issues and expected to increase enormously in the United States during the next 30 years (Pahwa et al., 2020). Inflammation is the pathological response of the body towards the external stimuli such as infectious, chemical, mechanical, and autoimmune stressors. Depending on post inflammatory responses, inflammation may be acute or chronic. Acute inflammation concentrates immune cells at the site of infection to combat dangerous foreign material while chronic inflammation is defined by the type of inflammatory cells in tissues when acute inflammation persists for a longer time (Ward, 2010). Advances in molecular studies show that chronic inflammation causes diabetes, heart disease, cancer, stroke, arthritis, and obesity (Pahwa et al., 2020) (Figure 1). It should be noted that inflammation is a self-healing process that proceeds in three crucial steps which are interconnected and occur sequentially such as swelling, redness, immobility, pain, and heat (Yatoo et al., 2018). Firstly, it starts from an increased vascular permeability followed by infiltration of immune cells that finally results in granuloma formation and tissue repair (Eddouks et al., 2012). Activated immunogenic response triggers mitogen-activated protein kinase (MAPK), Janus kinase/signal transducers and activators of transcription (JAK-STAT), and nuclear factor-κB (NF-κB) pathways, as well as the production of inflammatory cytokines, such as tumor necrosis factor-α (TNF-α), interleukin (IL) 1β (IL-1β), and chemokines (Afonina et al., 2017). Cytokines and chemokines both are critical for attracted activating additional immune cells at infection site, such as circulating neutrophils that boost interferon γ (IFN-γ), proteases, and reactive oxygen species (ROS). Cytokines also increase cyclooxygenase-2 (COX-2) that promotes the synthesis of inflammatory prostaglandins (Gandhi et al., 2017). After removing the immunogenic factor, the immune system reprograms signaling pathways to resolve inflammation in a dynamic process regulated by several biological systems. First, deployed effector cells are killed and reduced to baseline levels following elimination of proinflammatory agents and signals. Non-inflammatory macrophages remove apoptotic neutrophil vesicles and restores tissue equilibrium (Maskrey et al., 2011). However, sometimes the underlying conditions of the body interrupts with this phenomenon and lead to dysregulation of the inflammatory system, resulting in uncontrolled pathways and the production of inflammatory mediators that cause chronic inflammation and other degenerative diseases. One evidence meets here with regards to a link between inflammation and obesity (Stepien et al., 2014). In the present review, we have postulated a basic understanding of inflammation, obesity and other related complications while more emphasized on recent investigations of medicinal phytochemicals for their anti-inflammatory properties using preclinical and clinical studies.

## 2 Inflammation, obesity and related complications

Chronic inflammation is a condition that typically lasts for a long time and is characterized by the presence of immune cells such as lymphocytes and macrophages along with the proliferation of blood vessels and connective tissues. One remarkable discovery postulated that obesity is the biggest cause of chronic inflammation, following severe disorders (Ellulu et al., 2017). World Health Organization (WHO) estimated that 1.9 billion people are overweight and 600 million are obese (World Health Organization, 2015). Obesity increases pro-inflammatory IL-6 and TNF-α levels and decreases anti-inflammatory hormone adiponectin (Stepien et al., 2014). The overexpressed pro-inflammatory cytokines are considered to be the link between obesity and inflammation and this sustained chronic inflammation is a strong risk factor for developing many metabolic disorders and cancer (Hotamisligil, 2006).

**FIGURE 2:**
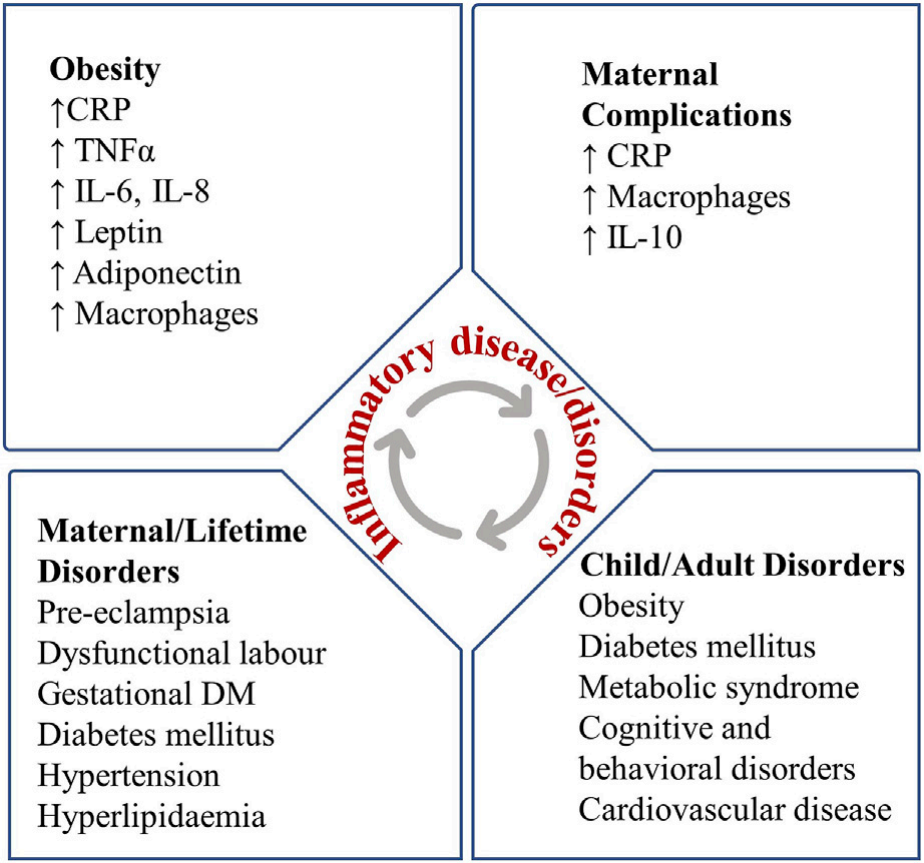
Relation between maternal obesity, inflammation and birth complications.

The adipose tissues are the determining factor of the occurrence of obesity. These tissues respond to additional nutrients by hyperplasia and hypertrophy, causing adipocyte expansion and obesity, which reduces blood flow and causes hypoxia (Cinti et al., 2005). Hypoxia is thought to cause necrosis and macrophage infiltration into adipose tissue, which leads to increased pro-inflammatory mediator production, including leptin, adiponectin, IL-6, TNF-α, monocyte chemoattractant protein-1 (MCP-1), and resistin (Lafontan, 2005). IL-6 induces hepatocytes to produce and release inflammatory molecules, c-reactive protein (CRP) that indicates liver-caused systemic inflammation which controls obesity regardless of race and gender (Choi et al., 2013). Klisic et al. (2014) measured CRP and metabolic markers among normal weight and overweight postmenopausal women and reported higher levels of CRP and triglycerides (TG) in overweight women. Adiponectin and leptin have a major role in inflammation; IL-6 also modulates the secretion of these hormones (Matsuda et al., 2002; Matsuzawa, 2006; Klisic et al., 2014). IL-6, adiponectin, leptin, and CRP are significant mediators of localized inflammation in adipose tissues when abnormalities are present. In this situation, obesity-related comorbidities develop, indicating an inflammatory state that contributes to the onset and progression of many diseases (Trayhurn and Wood, 2004; Hansson, 2005; Danesh et al., 2008; Zhang et al., 2009; Sansone and Bromberg, 2012) (Figure 2).

Obesity and inflammation have interrelated effects on the immune system, body weight, and metabolism (Castanon et al., 2014; McNelis and Olefsky, 2014). A study found a link between inflammation and ω-3 and ω-6 polyunsaturated fatty acids (PUFA) ratio. Larger consumption of ω-3 fatty acids reduces proinflammatory cytokines, IL-2, IL-6, and TNF-α, and increases anti-inflammatory IL-10 and tumor growth factor- β (TGF-β) (Alfano et al., 2012). High ω-6 PUFA diets increase adipokine levels, pro-inflammatory cytokine production, and hyperinsulinemia (Chan and Norat, 2015; Ghose et al., 2015). In animal studies, Polyak et al. (2014) found that chemokine fractalkine receptor knockout animals gained less weight and had less white adipose tissue than controls. These animals also had lower adipose MCP-1, IL-1α, and TNF-α levels (Polyak et al., 2014). IL-18 knockout animals fed a high-fat diet gained weight and burned less energy. Additionally, central IL-18 infusion reduced high-fat meal consumption, demonstrating that IL-18 can influence food intake centrally (Zorrilla and Conti, 2014). In conclusion, chemokine/cytokines, fractalkine, and IL-18 affect weight gain and metabolic diseases, indicating an interdisciplinary approach to inflammation and high-fat diet/obesity. The data also shows a link between obesity, diet, and chronic inflammation, which causes multiple diseases/disorders.

### 2.1 Birth complications

Preeclampsia (PE) has a global incidence of 2.16% during pregnancy (Abalos et al., 2014) and causes proteinuria, thrombocytopenia, renal insufficiency, and liver disease (Pennington et al., 2012; Abalos et al., 2014). In a healthy pregnancy, the processes that promote uteroplacental vascular remodeling can lead to placental ischemia after placental inflammation, which releases substances into the maternal circulation. These substances stimulate immune cells in the body’s periphery, especially T and B lymphocytes, which cause endothelial cell dysfunction, vascular dysfunction, and high blood pressure (LaMarca et al., 2013; Roberts, 2014). Since pro-inflammatory processes influence placental ischemia-induced hypertension, these mechanisms are likely amplified in obese people. Obesity before pregnancy is linked to high levels of pro-inflammatory cytokines in the placenta and circulating IL-6 throughout pregnancy. Overweight women have thicker placental blood vessel walls than normal-weight women (Roberts et al., 2011). Increased leptin gene expression may also contribute to PE (Lepercq et al., 2003; Iwagaki et al., 2004), decreased uterine natural killer cells (Parker et al., 2014), and increased CD4^+^ T cells (Wallace et al., 2001).

### 2.2 Cognitive and behavioral disorders

Obesity-related inflammation also affects the neonatal child and gives birth to neurological complications and brain disorders (Edlow, 2018). Thus, obesity-induced or direct inflammation during pregnancy make autism, schizophrenia, attention-deficit hyperactivity disorder and major depressive disorder more prevalent (Patterson, 2009; Knuesel et al., 2014; Estes and McAllister, 2016). Schizophrenia is characterized by delusions, hallucinations, disordered thinking, and cognitive impairment. Its prevalence rose from 13.1 million in 1990 to 20.9 million in 2016 (Charlson et al., 2018). Severe infections and autoimmune diseases may increase the lifetime risk of schizophrenia and schizophrenia spectrum disorders (Meyer, 2011; Benros et al., 2014). In response to maternal inflammation, placental cytokines (IL-1, IL-6, and interferon-γ) increase fetal oxidative stress (Meyer et al., 2009). This irreversible dysregulation affects brain growth and function and increases schizophrenia risk. Proinflammatory cytokine IL-6 may link maternal inflammation to fetal brain development and later psychopathology (Kohli et al., 2007; Buss et al., 2012). A recent study with 84 newborns used machine learning and resting-state functional magnetic resonance imaging. It showed that variations in maternal IL-6 concentrations across the course of pregnancy are associated with individual differences in functional brain networks in the neonatal period and relate to future working memory performance (Rudolph et al., 2018).

### 2.3 Cardiovascular diseases

Inflammation plays a key role in atherosclerosis, which raises risk of cardiovascular diseases (CVD) (Steinberg, 2006). Atherosclerosis begins with low-density lipoproteins (LDL) build up in abnormally permeable artery endothelium. Overexpression of IL-6 in atheromatous fatty streaks, endothelium, smooth muscle, and adipose tissue accelerates atherosclerosis (Szekanecz et al., 1994; Hlatky et al., 2009). TNF-α plays a role in endothelial dysfunction, vascular dysregulation, monocyte adherence to endothelial cells, vascular oxidative stress, apoptosis, and the atherogenic response, which lead to thrombosis and coagulation (Ueland et al., 2012; Zhang and Zhang, 2012). Leptin and adipokine influence atherosclerosis after CVD (Chen et al., 2003; Sierra-Johnson et al., 2007). Obesity is a risk factor for endothelial dysfunction-related cardiovascular diseases like arterial hypertension and atherosclerosis. Adipokines affect triglyceride metabolism and adipocyte hypertrophy, which can lead to macrophage expansion in adipose tissue, inflammation, and increased production of proinflammatory cytokines TNF-α and IL-6 (Samad et al., 1997; Fried et al., 1998; Mahabadi et al., 2009). Increased macrophages and local inflammation may cause obesity-related metabolic dysfunctions like systemic inflammation and atherosclerosis.

### 2.4 Osteoarthritis

Arthritis is another chronic inflammatory condition that causes disability and pain and hinders socioeconomic life. Osteoarthritis (OA) affects 250 million people worldwide, mostly the elderly (Kotti et al., 2014). Cartilage degeneration, subchondral bone remodeling, osteophyte production, and synovium and joint capsule inflammation characterize OA (Goldring and Goldring, 2010). Numerous soluble mediators, like cytokines or prostaglandins, can stimulate chondrocyte matrix metalloproteinases (MMP) synthesis, causing inflammation. OA causes an imbalance between pro-inflammatory and anti-inflammatory cytokines in the synovium (Kulkarni et al., 2021). Osteophytes are pro-inflammatory due to high mast cell activity (Kulkarni et al., 2022). Once thought to be cartilage-driven, OA is characterized by inflammatory synovium (Goldring and Otero, 2011; Kapoor et al., 2011; Loeser et al., 2012). In obese people, obesity may link OA and inflammation where obese people have twice the risk of OA as normal-weight people (Yusuf et al., 2010). Obesity imbalances adipokines and other cytokines, which may cause osteoarthritis (Gomez et al., 2011). White adipose tissue is the most common source of adipokines, but the knee’s infrapatellar fat pad also may produce inflammatory mediators like IL-6, TNF-α, adipsin, adiponectin, and visfatin that reach the synovium and cartilage (Clockaerts et al., 2010; Klein-Wieringa et al., 2011).

### 2.5 Diabetes

The International Diabetes Federation (IDF) predicts 578 million cases of diabetes by 2030 and 700 million by 2045 (International Diabetes Federation, 2019). Diabetes is characterized by impaired glucose tolerance and hyperglycemia caused by insulin deficiency or resistance (Blair, 2016). Type 1 diabetes is caused by β-cell death due to autoimmune disorder whereas type 2 diabetes (T2DM) is linked to genetics, ethnicity, age, overweight, unhealthy diet, and lack of exercise. Growing evidence suggests these causal variables follow the same inflammatory pathways as a shared pathogenetic mediator in diabetes progression (Shoelson et al., 2006). Diabetes etiology, relationship with obesity, and biological function of adipose tissue are studied extensively. The amount of inflammatory factors produced by adipose tissue macrophages defines obesity (Weisberg et al., 2003; Xu et al., 2003). When macrophages and immune cells move into adipose tissue, they cause chronic low-grade inflammation. The latter produces TNF-α, IL-1, IL-6, IL-10, leptin, adiponectin, MCP, angiotensinogen, resistin, and other cytokines and chemokines (Kanda et al., 2006; Shoelson et al., 2007; Antonopoulos et al., 2015) that serve as the pathologic link between obesity, insulin resistance and diabetes (Nikolajczyk et al., 2011).

### 2.6 Cancer

Lifestyle and environmental factors, rather than inherited genetic defects, regulate the development of 90%–95% of all cancers (Aggarwal et al., 2009). Chronic inflammation produces reactive oxygen species (ROS) leading to mutations and proliferation of the pro-cancerous cells. Cancer-promoting cytokines like IL-6, IL-11, TNF-α, IL-1β, and IL-23 vary by tumor type and stage. Thus, inflammation is a central component of tumor development and progression. In tumor microenvironments, inflammatory cells and mediators promote proliferative signaling, migration, metastasis, and blood vessel growth (Anand et al., 2008; Hanahan and Coussens, 2012). Inflammation accelerates many phases of metastasis, a key factor in cancer mortality (Hanahan and Weinberg, 2011). One recent study has estimated that 3.6% of all new cancer cases worldwide are attributable to excess adiposity and that uterine, postmenopausal breast, and colon cancer account for 63.6% of cancers attributable to high body mass index (BMI) (Arnold et al., 2015). As obesity-induced chronic inflammation is a cancer precondition, it increases cancer incidence and death. Obesity modifies release of adipokines and cytokines, affecting many systemic processes, including the tumor environment. Adiponectin, leptin, IL-6, TNF-α, YKL-40 (chitinase-3-like-protein-1), osteopontin, and plasminogen activator inhibitor-1 (PAI-1) are all produced by adipocytes and stimulate cancer growth, progression, and metastasis (Quail and Dannenberg, 2019).

In summary, obesity and inflammation are two sides of the same coin; it doesn’t matter which comes first. Both conditions are subjected with one causing the other and give rise to multiple health complications. Moreover, the facts about inflammation-related diseases and disorders, with an emphasis on obesity, show that chronic inflammation is the main cause of these complications. The information on diseases associated with inflammation demonstrates that chronic inflammation is the primary outcome of these complications. Our immune effector cells produce ROS and cytokines that trigger paracrine and autocrine inflammation. Unchecked oxidative stress can cause inflammation and tissue damage (Bennett et al., 2018). Chemically synthesized drugs can treat these inflammatory complications. Two drug classes 1) Steroid-based anti-inflammatory drugs (SAIDs) and 2) Non-Steroidal Anti-Inflammatory Drugs (NSAIDs) were developed to overcome the side effects and limitations of steroidal anti-inflammatory drugs (Celotti and Laufer, 2001; Rainsford, 2007). Even though high-class drugs are available, there are cost, availability and most importantly, side effect restrictions. To address these disadvantages, medicines must target underlying inflammation to make therapeutic advances with no or fewer adverse effects. Since inflammation is complex, it requires multidimensional treatment. In this regard, medicinal herbs are gaining importance to prevent and treat inflammatory disorders. In traditional use, clinical trials, and experimental studies, multiple plants have shown anti-inflammatory effects (Arulselvan et al., 2016; Allegra, 2019).

## 3 Plant derived drugs: a historical perspective

Historical observation of folklore medicines reveals Ayurveda and herbalism with ancient plant uses (4500 BC) (Karunamoorthi et al., 2013). Herbal medicine is the practice of treating disease with plants, plant extracts, herbal preparations, and finished herbal products called phytomedicines that contain phytochemicals as active ingredients (Pan et al., 2014). Traditional Chinese, Indian, and Arabic herbal medicine are the three main herbal treatment systems today. Archaeological evidence shows that Iraq and China have used herbal medicine for 6,000 and 8,000 years ago, respectively (Leroi-Gourhan, 1975; Pan et al., 2014). The earliest records of natural products are from Mesopotamia (2600 B.C.), where clay tablets documented the use of oils derived from *Commiphora* species (myrrh) and *Cupressus sempervirens* L. (Cypress) to treat coughs, colds, and inflammation (Cragg and Newman, 2005). In the past 40 years, both developing and developed countries have used more herbs and herbal products for health. Aspirin, or acetylsalicylic acid (*Salix alba* L., White willow), is a well-known anti-inflammatory drug. Other important drugs include morphine and codeine (*opium poppy*), digitoxin (lady’s glove), anti-malarial quinine, and Pilocarpine (*Pilocarpus jaborandi* Holmes, Pilocarpus) (Tarver, 2014). With advances in technology and chemical sciences, herbal active ingredients are being isolated and studied for pharmacological uses. This revolution in phytopharmacology has led to the development of various phytomedicines. Table 1 lists plant-based chemicals that have been shown to treat illness.

**TABLE 1:** Plant derived drugs for commercial use in various diseases.

Drug	Class of drug	Plant source	Disease	References
Paclitaxel	Taxanes	*Taxus brevifolia*	Breast cancer	Cragg (1998)
Ingenol 3-*O*-angelate	Polyhydroxy diterpenoid	*Euphorbia peplus*	Skin cancer	Kedei et al. (2004); Ogbourne et al. (2004)
PG490-88(14-succinyl triptolide sodium salt)	Diterpene-diepoxide	*Tripterygium wilfordii*	Autoimmune and inflammatory diseases	Kiviharju et al. (2002); Fidler et al. (2003)
Tiotropium	Muscarinic receptor antagonist	*Atropa belladonna*	Asthma and COPD	Kumar and Reddy (2003)
Arteether	Sesquiterpene lactones	*Artemisia annua*	Antimalarial	Newman and Cragg (2007)
Grandisines A and B	Indole alkaloids	*Elaeocarpus grandis*	Analgesic	Carroll et al. (2005)
Galantamine hydrobromide	Amaryllidaceae alkaloid	*Galanthus nivalis*	Alzheimer’s	Howes et al. (2003)
Apomorphine	Dopamine	*Papaver somniferum*	Parkinson’s	Deleu et al. (2004)

## 4 Phytochemicals evaluated in anti-inflammatory properties

Increasing knowledge of folklore medicinal plants as a therapeutic target opened the door for anti-inflammatory plant extracts. Polyherbal formulation of *Ashwagandharishta*, *Balarishta*, *Dashmoolarishta*, and *Triphal*a extract reduces synovial inflammation (Ingale et al., 2018). Pawar et al. (2011) tested *Withania somnifera* L. root extracts in an inflammatory bowel disease (IBD) rat model (Pawar et al., 2011). *Piper ovatum* Vahl leaves have been examined for their anti-inflammatory properties by Rodrigues Silva et al. (2008). Ayurveda describes fermented Asava and Arishta formulations. These formulations are plant extracts fermented with microbes, allowing biological transformation and potentially generating novel fermentative products of phytochemicals with superior bioavailability and anti-inflammatory activity (Bhondave et al., 2014). Carrageenan-injected rats showed anti-inflammatory effects from *Eulophia ochreata* L. tubers extract (Jagtap et al., 2009). An animal model of carrageenan-induced inflammation was used to test the anti-inflammatory properties of the ethanolic root extract of *Swertia chirata* Buch.-Ham. ex Wall (Das et al., 2012). To understand the plant’s anti-inflammatory role and mechanism, researchers are isolating and characterizing phytochemicals and organizing them by structure and chemical properties. Understanding phytochemical mechanisms of action could lead to new anti-inflammatory drugs.

## 5 Preclinical trials

**FIGURE 3:**
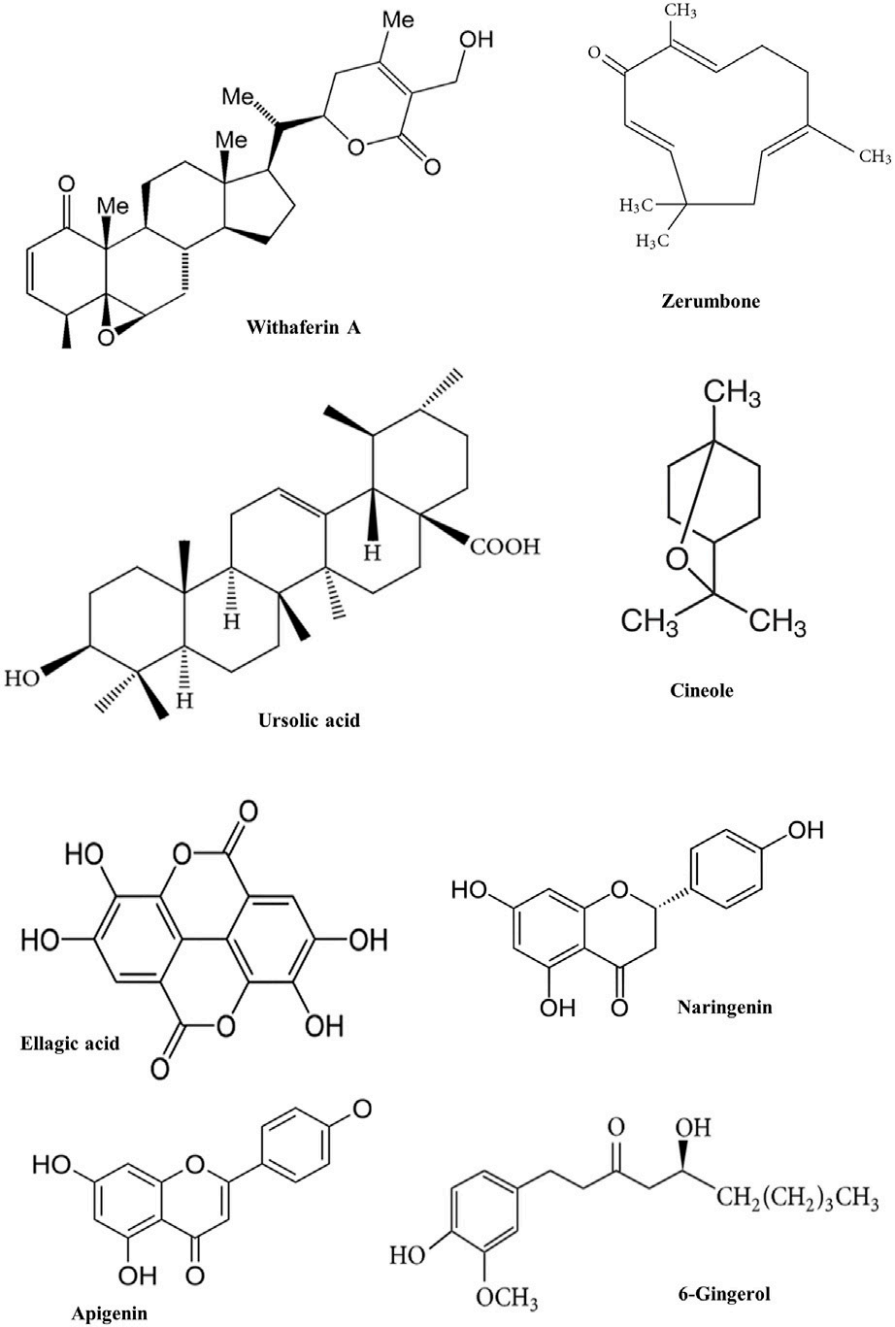
Chemical structure of some phytochemicals used in pre-clinical trials.

First, phytochemicals are tested *in vitro*, then *in vivo* using animal models, and finally in humans. Selecting the right experimental model prevents bias and errors. This study examined *in vitro* and *in vivo* anti-inflammatory phytochemicals and plant-based anti-inflammatory drug possibilities. In this section, potential phytochemicals (Figure 3) studied for anti-inflammatory diseases/complications in preclinical experiments are discussed (Tables 2, 3).

**TABLE 2:** List of anti-inflammatory phytochemicals used in pre-clinical trials.

Class	Phytochemicals	Botanical name (family)	Molecular targets	References
*Flavones*	Apigenin	*Chamaemelum nobile* (Asteraceae)	NF-κB and STAT 1 Signalling pathways, expression of miR-155, activation of PPAR-γ	Nicholas et al. (2007), Arango et al. (2015), Feng et al. (2016)
	Luteolin	*Rosmarinus officinalis* (Lamiaceae)	JNK, NF-κB p65 Signalling pathways	Ando et al. (2009), Xiong et al. (2017)
	Baicalin and Baicalein	*Scutellaria baicalensis* (Lamiaceae)	Fizz1 expression, CHOP/STAT pathway	Kim et al. (2018), Zhu et al. (2016)
*Flavonols*	Quercetin	*Malus domestica* (Rosaceae)	NF-κB p65, ERK, JNK and STAT pathways, AKT Signalling modulation	Bian et al. (2018), Maurya and Vinayak, (2017)
	Kaempferol	*Camellia sinensis* (Theaceae)	NF-κB, STAT, and JNK Signalling pathways	Bian et al. (2019), Park et al. (2015)
*Flavanone*	Naringenin	*Citrus paradise* (Rutaceae)	NF-κB activation, NF-kB/IL-6/STAT-3 pathways, NO-cGMP-PKG KATP channel Signalling	Pinho-Ribeiro et al. (2016), Zhang et al. (2018b), Manchope et al., (2016)
	Hesperidin	*Citrus limon (*Rutaceae)	expression of p65, Foxo1, Foxo3, and Nrf2 Signalling pathways	Xiao et al. (2018), Tsai et al. (2019)
*Isoflavones*	Genistein	*Genista tinctorial* (Fabaceae)	NF-κB/Akt Signalling pathway, AMPK activation, expression of p65	Howes and Simmonds (2014), Lee et al. (2019), Yuan et al. (2019)
	Puerarin	*Pueraria lobate* (Fabaceae)	NF-κB activation, Fizz1 expression, Nrf2 regulation	Liu et al. (2014a), Nguyen Ngo Le et al. (2019), Jeon et al. (2020)
*Catechins*	Epigallocatechin gallate	*Camellia sinensis* (Theaceae)	Suppression of neuronal apoptosis, NF-κB/p65/IκB-α Signalling pathway	Cai et al. (2014)
*Anthocyanidins*	Cyanidin-3-O-glycoside	*Lonicera caerulea* (Caprifoliaceae)	MAPK and NF-κB Signalling pathway, regulation of iNOS and COX-2 expression	Wu et al. (2017), Pereira, Almeida, and Dinis (2018)
*Monoterpenes*	Cineole	*Eucalyptus globulus* (Myrtaceae)	PPAR-γ dependent modulation of NF-κB, PRR pathways, NF-κB/MAPKs/MKP-1 Signalling pathways	Linghu et al. (2019), Yadav and Chandra (2017)
	Paeoniflorin	*Paeonia lactiflora* (Paeoniaceae)	Nrf2/HO-1 Signalling pathways, MAPK pathway, ERK1/2 and Akt regulation, NF-κB/p65/IκBα signalling pathways	Wu et al. (2019), Yu et al., (2017), Gong et al. (2015), Yu et al. (2019)
*Sesquiterpenes*	Parthenolide	*Tanacetum parthenium (*Asteraceae)	NF-κB and MAPKs signalling pathways Nrf2/Keap1 signalling pathway	Kim et al. (2019), Li et al. (2015)
	Zerumbone	*Zingiber zerumbet* (Zingiberaceae)	NF-κB/HO-1 signalling pathway, NF-κB/MAPK/PI3K-Akt signalling pathways	Kim et al. (2009), Haque et al., (2018)
*Diterpenoids*	Ginkgolides	*Ginkgo biloba* (Ginkgoaceae)	Regulation of Caspase-1/NF-κB P65 expression, CD40-NF-κB signal pathway	Chen et al. 2018b), Zhang et al. (2018)
*Triterpenoids*	Ursolic acid	*Glechoma hederacea* (Lamiaceae)	NF-κB/p65 signalling pathway	Zhao et al. (2018)
	Escin	*Aesculus hippocastanum,* (Sapindaceae)	mRNA expression of NF-κB/reduction of TNF-α, P-selectin, and VCAM-1	Wang et al., (2014), Zhao et al. (2018)
	Withaferin A	*Withania somnifera* (Solanaceae)	IKKβ/NF-κβ pathway, regulation of LPS/TLR4 pathway	Martorana et al., (2015), Batumalaie et al., (2016)
	β –sitosterol	*Glycine* max (Fabaceae)	SHP-1/NF-κB regulation, NLRP3/caspase-1 signalling pathway	Valerio and Awad (2011), Liao et al., (2018)
*Curcuminoids*	Curcumin	*Curcuma longa*, (Zingiberaceae)	TLR4/MyD88/NF-κB signalling pathway, PI3K/Akt/NF-κB signalling pathway, NF-κB/PPAR-γ signalling, MAPK/ERK/p38/Akt/NF-κB pathway, HO-1, and Nrf-2 pathway	Zhu et al. (2014), Song et al., (2013), Liu et al., (2016b), Yu et al., (2018)
*Stilbenes*	Resveratrol	*Vitis vinifera* (Vitaceae)	Modulation of AP-1/NF-κB/COX-2, ICAM-1, iNOS, and IL-1β mRNA expression, VEGF/p38-MAPK/NF-κB pathway	Latruffe et al., (2015), Huang et al., (2017), Yan et al., (2018)
*Phenolic acids*	Rosmarinic acid	*Rosmarinus officinalis* (Lamiaceae)	NF-κB and p65 expression, NF-κB/p65/pSTAT3 pathway	Cao et al., (2016), Jin et al., (2017)
	Ellagic acid	*Punica granatum* (Lythraceae)	expression of RANTES protein, IRAK4/TRAF-6/IKK-β/NF-κB/p65 expressions	(Promsong et al., (2015), Zhou et al., 2019)
	Gallic acid	*Camellia sinensis* (Theaceae)	TLR-4/NF-κB/PPARγ signalling pathway	Fan et al. (2018)
	Protocatechuic acid	*Allium cepa* (Amaryllidaceae)	SIRT1/NF-*κ*B signalling pathway PI3K/Akt-mediated nuclear-factor-κB activation, STAT-6/PPAR-γ pathway	Kaewmool et al. (2020)
	Vanillic acid	*Vanilla planifolia* (Orchidaceae)	Nrf2/HO-1 expression	Calixto-Campos et al. (2015)
	6-gingerol	*Zingiber officinale* Rosc. (Zingiberaceae)	PI3K and p-Akt expression, RANKL/PGE2 expressions	Xu et al. (2018b), Hwang et al. (2018)
	Caffeic acid phenethyl ester	*Populus nigra* L. (Salicaceae)	NF-κB/p65 signalling pathway, Nrf2/HO-1 signalling pathway	Takakura et al. (2018)

**TABLE 3:** List of some animal models with the target pathologies in pre-clinical trials.

Phytochemicals	Animal models	Target pathologies	References
Apigenin	Male C57BL/6J	Colonic inflammatory and motor abnormalities	Gentile et al. (2018)
Luteolin	C57BL/6J Obese mice model	Insulin resistance (IR) and type 2 diabetes pathophysiology	Liu et al. (2014b)
Baicalin and Baicalein	LPS-induced mastitis in BALB/c mice	Mastitis	He et al. (2015)
Quercetin	Male C57BL/6N mice	Angiogenesis in lymphoma-bearing mice	Khan et al. (2018)
Kaempferol	BALB/c mice models	Allergic asthma	Park et al. (2015)
Naringenin	Male Swiss mice	Superoxide anion-driven inflammatory pain	Manchope et al. (2016)
Hesperidin	Sprague-Dawley rats	Diabetic neuropathy	Visnagri et al. (2014)
Genistein	Diethyl nitrosamine induced C57BL/6 N mice	Hepatocellular carcinoma	Lee et al. (2019)
Puerarin	Male Sprague-Dawley rats	Streptozotocin (STZ)-induced diabetes	Liu et al. (2014a)
Epigallocatechin gallate	Male adult Sprague–Dawley (SD) rats	Chronic constriction injury	Cai et al. (2014)
Cyanidin-3-O-glycoside	TNBS-challenged mice	Inflammation in colitis	Gan et al. (2019)
Cineole	Male Kunming mice	LPS-induced acute inflammatory injury	Linghu et al. (2019)
Paeoniflorin	Adult male Sprague-Dawley rats	Chronic constriction injury	Zhou et al. (2019b)
Parthenolide	Collagen antibody-induced arthritis (CAIA) BALB/c mouse model	Rheumatoid arthritis	Williams et al. (2020)
Zerumbone	Mono-iodoacetate (MIA)-induced male SD rat OA model	Osteoarthritis	Chien et al. (2016)
Ginkgolides	Male Sprague-Dawley rats	Myocardial ischemia/reperfusion	Zhang et al. (2018a)
Ursolic acid	1-methyl-4-phenyl-1,2,3,6-tetrahydropyridine (MPTP)-intoxicated Parkinson mouse model	Neuroinflammation	Rai et al. (2019)
Escin	Swiss albino mice	Indomethacin-induced gastric ulcers	Wang et al. (2014)
Withaferin A	BALB/c mice	Spinal cord tissues in traumatized mice	Yan et al. (2017)
β –sitosterol	CIA mice model	Arthritis	Liu et al. (2019a)
Curcumin	BALB/c mice	Allergic asthma	Shahid et al. (2019)
Resveratrol	Cigarette smoke COPD mouse models	Chronic obstructive pulmonary disease	Chen et al. (2016)
Rosmarinic acid	DSS-induced colitis mouse model	Splenomegaly	Jin et al. (2017)
Ellagic acid	streptozotocin-induced diabetic nephropathy mouse model	Diabetic Nephropathy	Zhou et al. (2019a)
Gallic acid	sulfonic acid (TNBS)-induced ulcerative colitis (UC) mouse model	Ulcerative colitis	Zhu et al. (2019)
Protocatechuic acid	castrated rats	Benign prostatic hyperplasia	Akanni et al. (2020)
Vanillic acid	carrageenan-induced inflammatory pain mouse model	Analgesic and anti-inflammatory effects	Calixto-Campos et al. (2015)
6-gingerol	Sprague-Dawley rats	myocardial infarction	Xu et al. (2018b)
Caffeic acid phenethyl ester	rat model of optic nerve crush (ONC) injury	Glaucoma	Takakura et al. (2018)

### 5.1 Flavones

#### 5.1.1 Apigenin (APG)

Apigenin (APG) is found in *Chamaemelum nobile* (L.) All. (Asteraceae) ligulate flowers, celery, parsley, coriander, and peppermint. Anti-inflammatory activity of APG involves inhibiting of NF-κB translocation by suppressing p65 phosphorylation (Nicholas et al., 2007). In an IFN-γ activated murine microglia cell model, APG’s effect on STAT1 phosphorylation reduced IL-6 and TNF-α levels (Rezai-Zadeh et al., 2008). APG and APG-rich diets may have anti-inflammatory effects *in vivo* by lowering lipopolysaccharide (LPS)-induced microRNA-155 (Arango et al., 2015). Diet-induced obesity in male C57BL/6J mice was used to study APG’s effects on inflammatory and motor abnormalities in the colon. APG (10 mg/kg) stopped the increase in body fat, epididymal fat, and metabolic indexes. There was also a reduction in malondialdehyde (MDA), IL-1β, IL-6, eosinophil infiltration, substance P, and inducible nitric oxide synthase (iNOS expression) (Gentile et al., 2018). Alzheimer’s, Parkinson’s, and Huntington’s are neurodegenerative diseases caused by neuroinflammation. APG showed strong anti-inflammatory properties in a human-induced pluripotent stem cell (iPSC) model of familial and sporadic Alzheimer by protecting neurites and cell viability by downregulating cytokine and nitric oxide (NO) release in inflammatory cells (Balez et al., 2016). Non-alcoholic steatohepatitis (NASH) causes a fatty, inflamed liver. APG (0.005%, w/w) reduced inflammation by lowering plasma MCP-1, IFN-γ, TNF-α, and IL-6 levels in mice with NASH and a high-fat diet (Jung et al., 2016). In diabetic rats, APG (10, 30, 50 mg/kg) reduced metabolic inflammation by successfully polarizing infiltrating macrophages to an anti-inflammatory M2 phenotype. The mechanism involved binding and activating peroxisome proliferator-activated receptor gamma (PPAR-γ) and the subsequent suppression of the NF-κB pathway (Feng et al., 2016).

#### 5.1.2 Luteolin

This is a common flavone found in rosemary (*Rosmarinus officinalis* L., Lamiaceae), pomegranate (*Punica granatum* L., Lythraceae) and artichoke (*Cynara scolymus* L., Asteraceae). Luteolin suppresses chronic inflammation in adipocytes and macrophages coculture, as well as c-Jun N-terminal Kinase (JNK) phosphorylation in macrophages (Ando et al., 2009). In the C57BL/6J obese mice model, luteolin (10 mg/kg) reduces MCP-1 and resistin in blood, while elevated adiponectin level that improved insulin resistance (IR) and T2DM (Liu et al., 2014b). Multiple sclerosis (MS), a neurodegenerative and immune-inflammatory disorder, causes problems throughout the body. Immunomodulatory effects on peripheral blood mononuclear cells (PBMC) derived from MS patients were observed in the presence of luteolin where it suppressed pro-inflammatory cytokines, including IL-1β, MMP-9, and TNF-α (Sternberg et al., 2009). The effects of luteolin were also examined on irinotecan-induced mice model of intestinal mucositis. It reduced ROS levels and inflammation by lowering TNF-α, IL-1β, and IL-6 whereas increased the levels of IL-4 and IL-10 (Boeing et al., 2020). Severe acute pancreatitis (SAP) is pancreatic inflammation and the outcome may be life-threatening. Xiong et al. (2017) studied the effects of luteolin in an ICR mouse model induced by cerulein/LPS where luteolin (100 mg/kg) reduced SAP symptoms by lowering TNF-α and IL-6 levels while raising IL-10 via NF-κB p65 and IκBα expressions (Xiong et al., 2017). In a study, skin from BALB/c mice donors was grafted in C57BL/6 mice recipients and allografts were treated with luteolin (25 and 50 mg/kg). The recipient mice survived longer showing decreased cellular infiltration and proinflammatory cytokine gene expression (Ye et al., 2019).

#### 5.1.3 Baicalin and baicalein

*Scutellaria baicalensis* Georgi (Lamiaceae) is a traditional Chinese herb that contains the compounds baicalin and baicalein. IBD is a long-term, idiopathic inflammation which causes small and large intestine complications. Zhu et al. (2016) studied the baicalin (100 mg/kg) effects on macrophage polarization and IBD therapy. He found that LPS-stimulated mouse peritoneal macrophages had a lower ratio of M1 to M2 macrophages, indicating a shift from M1 to M2 polarization, especially Fizz1 expression in M2a subtypes. Baicalin has also been found effective in colitis, an auto-immune or infectious colon inflammation. A report suggested that baicalin upregulated both interferon regulatory factor 4 and 5 in lamina propria mononuclear cells isolated from dextran sulfate sodium (DSS)-induced colitis mice model (Zhu et al., 2016).

Multiple studies are also present that emphasize the anti-inflammatory properties of baicalein. Kim et al. (2018) showed that baicalein blocks NO, cytokines, chemokines and growth factors through the endoplasmic reticulum stress CHOP/STAT pathway in RAW 264.7 murine macrophages induced by dsRNA (Kim et al., 2018). Tubular-interstitial nephritis is characterized by kidney inflammation and cell damage. A report suggested that baicalein alleviated LPS induced cell viability and apoptosis of renal tubular epithelial cells, while decreased the activation of NF-κB and MAPKs (Chen et al., 2018a). Hepatic ischemia/reperfusion (I/R) is an inflammatory liver pathology. It was found that baicalein (300 mg/kg) preconditioning reduced NF-κB expression and pro-inflammatory cytokine production whereas TNF-α/IL-10 ratio and leukocyte infiltration were reduced (Mahmoud et al., 2019). Furthermore, in a report, baicalein (20 mg/kg) consistently suppressed T-cell proliferation in collagen-induced C57BL/6J male mice of arthritis (CIA) (Xu et al., 2018a). Mastitis is a breast inflammation which is usually caused by a bacterial infection. In BALB/c mice with LPS-induced mastitis, baicalein (20 mg/kg) reduced mammary gland damage, myeloperoxidase activity, TNF-α and IL-1β levels, while blocked the TLR4 expression. Baicalein suppressed TLR4-mediated NF-κB and MAPK signaling, reducing inflammation (He et al., 2015).

### 5.2 Flavonol

#### 5.2.1 Quercetin

Quercetin is a common flavonol found in fruits and vegetables (*Malus domestica* Borkh., Rosaceae). Activated endothelial cells control leukocyte trafficking to inflammation sites in early atherosclerosis. One report found that quercetin reduced COX, 5-LOX 9 (arachidonate 5-lipoxygenase), MPO, NOS, CRP, and IL-6 mRNA expression in Sprague-Dawley (SD) rats on a hypercholesterolemic diet (Bhaskar et al., 2016). Interstitial inflammation is the primary pathogen following a kidney insult, as inflammatory macrophages become polarized. Quercetin (20 mg/kg) reduced tubulointerstitial damage and inflammatory factor production in ICR/JCL mice with obstructed kidneys while CD68^+^ macrophages infiltrated the renal interstitium less often. Reduced iNOS and IL-12 levels and increased F4/80^+^/CD11b^+^/CD86^+^ macrophages in kidneys of renal injury patients suggested quercetin prevented M1 macrophage polarization (Lu et al., 2018). Inflammation in IBD requires activated microvascular endothelial cells and cell adhesion. In LPS-stimulated rat intestinal microvascular endothelial cells, quercetin reduced intercellular adhesion molecules (ICAMs) and vascular cell adhesion molecule-1 (VCAM-1) protein levels. This phytochemical reduced TLR4, NF-κB p65, extracellular signal-regulated kinase (ERK), JNK, STAT phosphorylation and IκB-α degradation (Bian et al., 2018). AKT (protein kinase B) signaling is often activated in cancer, which keeps the tumor microenvironment oxidized for adaptability. A report found that quercetin reduced cell survival, inflammation, and angiogenesis in lymphoma-bearing mice (Maurya and Vinayak, 2017). Khan et al. (2018) explained that quercetin (30 mg/kg/day) reduced activated gliosis and inflammatory markers and stopped neuroinflammation in adult male of C57BL/6N brain and hippocampal regions (Khan et al., 2018).

#### 5.2.2 Kaempferol

It is a flavonoid found in tea [*Camellia sinensis* (L.) Kuntze, Theaceae] and many fruits and vegetables (also known as kaempferol-3 or kaempferide). Intervertebral disc degeneration has been considered an irreversible process when cell viability decreases, type II collagen is synthesized and the nucleus pulposus is dehydrated. Research proved that in the presence of Kaempferol, proinflammatory cytokines decreases while IL-10 increases (Zhu et al., 2017). Wang et al. (2018) reported that kaempferol suppressed concanavalin A-induced T-cell proliferation and NO/ROS generation in LPS-infected RAW 264.7 macrophage cells (Wang et al., 2018). It is known that endothelial expression of cytokines and adhesion molecules triggers IBD. One report emphasized the role of kaempferol where it stopped rat intestinal microvascular endothelial cells from making too much TNF-α, IL-1β, IL-6, ICAM-1, and VCAM-1 via NF-κB and STAT signaling pathways (Bian et al., 2019). The NF-κB pathway, is critical in inflammation, proliferation, and carcinogenesis. Kaempferol reduced NF-κB activity in secreted embryonic alkaline phosphatase (SEAP)-driven NF-κB reporter cells with varying TNF-α concentrations (Kadioglu et al., 2015). Allergic asthma is a respiratory condition which causes airway inflammation. kaempferol (20 mg/kg) reduced allergic asthmatic mucus production in BALB/c mice by disrupting TGF-β-triggered ER stress signaling of inositol-requiring enzyme 1α/TNF receptor-associated factor 2/c-Jun N-terminal kinase (Park et al., 2015).

### 5.3 Flavanones

#### 5.3.1 Naringenin

Grapefruits contain bitter, colorless flavonoid naringenin (*Citrus paradisi* Macfad., Rutaceae) which is known to reduce inflammatory and nerve pain. It was reported that oxidative stress, hyperalgesic cytokines (IL-33, TNF-α and IL-1β), and NF-κB activation were inhibited in mice paw skin treated with naringenin (16.7–150 mg/kg) (Pinho-Ribeiro et al., 2016). Naringenin also reduced colitis by inhibiting myeloid-derived suppressor cells, pro-inflammatory mediators, and the NF-κB/IL-6/STAT-3 cascade in colonic tissues (Zhang et al., 2018). Naringenin’s anti-inflammatory and anti-allergy properties were tested on mice models of ear edema caused by arachidonic acid and tetradecanoylphorbol-13-acetate (TPA). Naringenin showed anti-inflammatory effects against otitis media in female CD-1 mice at 1% in arachidonic acid and 50% in TPA (Escribano-Ferrer et al., 2019). Narringenin (50 mg/kg) reduces nociceptive effects and inflammation in male Swiss mice by activating the NO-cGMP-PKG-KATP channel signaling pathway involving nuclear factor erythroid 2-related factor 2/heme oxygenase-1 (Nrf2/HO-1) (Manchope et al., 2016).

#### 5.3.2 Hesperidin

Flavonoid hesperidin is found in citrus fruits, especially oranges and lemons (*Citrus limon* (L.) Osbeck, Rutaceae). Diabetic neuropathy (DN) is one of the most common long-term complications of diabetes mellitus. In the diabetic neuropathy model of SD rats, hesperidin (50 and 100 mg/kg) reduced IL-1β and TNF-α (Visnagri et al., 2014). Moreover, hesperidin effectively enhanced chondrogenesis of human mesenchymal stem cells (MSCs) by inhibiting pro-inflammatory cytokines IFN-γ, IL-2, IL-4 and IL-10, and suppressing the expression of p65 to facilitate cartilage tissue repair (Xiao et al., 2018). Oxidative stress can cause chondrocytes to secrete inflammatory mediators, causing a senescence-associated secretory phenotype. Hesperidin showed chondroprotective properties, increased cellular antioxidant capacity, decreased COX-2, IL-1β, TNF-α, MMP-3, MMP-9 mRNA levels, and increased IL-10, tissue inhibitors of metalloproteinases-1, SRY-box transcription factor 9, and altered forkhead box O 1 (Foxo1), Foxo3, and Nrf2 signaling pathways in H_2_O_2_ stimulated primary human chondrocytes (Tsai et al., 2019). OA is one of the degenerative and chronic diseases of articular joints with chondrocytes degeneration. Hesperidin reduces IL-1β-induced MMP-3 and MMP-13 expression in OA chondrocytes and NF-κB (Fu et al., 2018). Hesperidin (100 mg/kg) inhibited inflammation in an Alzheimer’s disease (AD) APP/PS1 mouse model, restored APP synthesis and Aβ peptide deposition, and improved nesting and social interactions (Li et al., 2015a).

### 5.4 Isoflavones

#### 5.4.1 Genistein

Genistein is an isoflavone polyphenol extracted from *Genista tinctorial* L., the dyer’s broom (Fabaceae). Genistein suppresses NF-κB activation, reduces TNF-α and IL-6 production, and reactivates insulin-mediated Akt and endothelial NO synthase phosphorylation to improve insulin resistance-related endothelial dysfunction. Endothelin-1, a cytokine that plays a role in insulin’s mitogenic effects, was also downregulated by the treatment and VCAM-1 overexpression (Howes and Simmonds, 2014). Genistein also inhibited NO, Prostaglandin E2 (PGE2), IL-1, TNF-α, TLR4 and MyD88 in LPS-induced BV2 microglia (Jeong et al., 2014). It has been evidenced that chronic inflammation develops hepatocellular carcinoma (HCC) and other malignancies. When C57BL/6N mice were given 80 mg/kg of Genistein, it slows down HCC development while AMP-activated protein kinase activation killed hepatocytes through caspase pathways and reduced liver macrophage inflammation (Lee et al., 2019). Breast cancer is the most common malignancy in women of developed countries. The effects of the phytoestrogen genistein on the inflammatory profile in breast cancer cell lines were studied. Genistein-dependent expression of inflammatory-related genes was seen through its interaction with alpha and beta estrogen receptors (ER), and its effects depend on the ERα/ERβ ratio (Pons et al., 2019). In experimentally induced condylar cartilage degradation in male rats, genistein (180 mg/kg) treatment had significantly reduced the expression of p65 and inflammatory cytokines (IL-1β and TNFα) showing therapeutic effects on condyle cartilage damages of OA rats (Yuan et al., 2019).

#### 5.4.2 Puerarin

Puerarin is a key component of *Pueraria lobata* (Willd.) Ohwi (Pueraria lacei Craib) (Fabaceae). Xue et al. (2016) reported that puerarin inhibited MDA, NO, NF-κB, TNF-α, IL-1β, and IL-6 production in an animal I/R model (Xue et al., 2016). In streptozotocin induced diabetic male SD rats, Puerarin reduced spinal cord inflammation and neuropathic pain by inhibiting NF-κB activation and cytokine upregulation (Liu et al., 2014a). A rat model (rAION) of anterior ischemic optic neuropathy was used to test puerarin’s antiapoptotic and anti-inflammatory effects. Anti-apoptotic factors were increased by reducing iNOS, IL-1β, TNF-α, and IL-10 and inducing IL-10, arginase-1, and Fizz1 (found in inflammatory zone protein) (Le et al., 2019). *In vitro* and *in vivo* OA models were used to study the therapeutic effects of puerarin. It increases OA chondrocyte proliferation and suppresses IL-1β induced inflammatory cytokines and monocytes/macrophages. In a mono-iodoacetate-induced OA mouse model, puerarin (50 mg/kg) reduced inflammatory monocyte recruitment and cartilage destruction (Peng et al., 2019). Ulcerative colitis is an IBD accompanied by abdominal pain, diarrhea, and rectal bleeding. Puerarin was given to male BALB/c mice with DSS-induced colitis at 10 and 50 mg/kg, where it showed antioxidant mechanism by controlling the Nrf2 pathway and antioxidant enzymes. It also inhibited NF-κB and pro-inflammatory mediators of inflammation (Jeon et al., 2020).

### 5.5 Catechins

#### 5.5.1 Epigallocatechin gallate (EGCG)

Green tea leaves (Camellia sinensis (L.) Kuntze, Theaceae) have the most EGCG catechins. Chronic constriction injury (CCI)-induced neuropathic pain in male adult SD rats are improved by intrathecal injection of EGCG (1 mg/kg), which reduces TLR4, NF-κB, High mobility group box 1, TNF-α, and IL-1β and increases spinal cord IL-10 (Kuang et al., 2012). Infrasound, a common source of vibroacoustic illness, can harm the central nervous system (CNS). EGCG inhibited infrasound-induced microglial activation in rat hippocampi, as evidenced by reduced expression of IL-1, IL-6, IL-18, and TNF-α cytokines and decreased neuronal apoptosis. EGCG reduced microglia IκBα and infrasound-induced nuclear NF-κB, p65, and phosphorylated IκBα (Cai et al., 2014). Sun et al. (2017) reported that EGCG improved renal pathology and reduced inflammatory markers in diabetic mice, including ICAM1 and VCAM-1 (Sun et al., 2017). EGCG (50 mg/kg) reduced macrophage and T-cell infiltration in Dahl salt-sensitive rats (Luo et al., 2020). In Balb/c mouse models with bronchial asthma, EGCG (20 mg/kg) reduces airway inflammation via the TGF-1β pathway and eventually reduced Th17 cells and increased Treg cells (Shan et al., 2018).

### 5.6 Anthocyanidins

#### 5.6.1 Cyanidin-3-O-glycoside (C3G)

C3G is a pigment in red and blue fruits and vegetables. *Lonicera caerulea* L contains anti-inflammatory anthocyanins (Caprifoliaceae). C3G inhibits the NF-κB pathway in epithelial cells, protecting against chronic gut inflammatory diseases (Ferrari et al., 2017). C3G may reduce LPS-induced inflammation through TAK1 (transforming growth factor-β-activated kinase 1) mediated MAPK and NF-κB pathways, according to a mouse paw edema and macrophage cell model (Wu et al., 2017). Researchers used an LPS-activated macrophage cell line (RAW264.7) to test C3G and 5-aminosalicylic acid’s anti-inflammatory properties. iNOS and COX-2 expression inhibition were more effective than 5-aminosalicylic acid at countering LPS-induced NO and prostaglandin release (Pereira et al., 2018). 2,4,6-trinitrobenzene sulfonic acid (TNBS)-induced colitis in mice and LPS-stimulated C3G and cyanidin were used to examine Caco-2 cell monolayer inflammation. Chronic exposure to TNBS reduced the animal’s clinical symptoms and histological brain damage. Activation of myeloperoxidase and release of inflammatory cytokines TNF-α, IL-1β, IL-6, and IFN-γ were dramatically reduced. Caco-2 cells treated with LPS produced less nitric oxide and inflammatory cytokines when C3G or Cy was added (Gan et al., 2019). Microglia are resident macrophages involved in many neurodegenerative diseasescause brain inflammation. Pre-treatment with C3G reduced microglial activation and the production of neurotoxic mediators like NO, PGE2, and pro-inflammatory cytokines (IL-1β and IL-6). C3G suppressed NF-κB and p38 MAPK signaling pathways, reducing iNOS, COX-2, and proinflammatory cytokines (Kaewmool et al., 2019).

### 5.7 Monoterpenes (terpenoids)

#### 5.7.1 Cineole

Cineole is also called eucalyptol or 1,8-cineole and the main volatile oil in *Eucalyptus* spp. (Myrtaceae). *In vitro* studies of normal and non-smoking monocytes showed IL-6 was inhibited more than IL-1β, IL-8, and TNF-α at 0.15–1.5 µM of 1,8-cineole (Juergens et al., 2017). 1,8-cineole protects vascular endothelium in LPS-induced mice, and human umbilical vein endothelial cells (HUVECs), inhibits IL-6 and IL-8 and boosts serum IL-10. Male Kunming mice given LPS had less inflammation and VCAM-1 expression in the thoracic aorta. *In vitro* and *in vivo* results showed that 1,8-cineole reduced LPS damage to endothelial cells through PPAR-dependent NF-κB modulation (Linghu et al., 2019). Eucalyptus oil, long used in traditional medicine, is helpful in aromatherapy for respiratory problems. Yadav et al. (2017) studied 1,8-cineole and eucalyptol regulate anti-inflammatory pathways by downregulating pattern recognition receptors (PRR) receptors (TREM-1 and NLRP3) and downstream signaling cascade partners (NF-κB, MAPKs, MKP-1) (Yadav and Chandra, 2017).

#### 5.7.2 Paeoniflorin (PF)

The main ingredient in *Paeonia lactiflora* Pall is paeoniflorin (PF) (Paeoniaceae). When LPS was added to Caco-2 cells, PF blocked COX-2, iNOS, TNF-α, IL-6, and MMP-9 and inhibited NF-κB signaling by activating Nrf2/HO-1 (Wu et al., 2019). It was shown that PF-treated psoriasis animal models had thinner epidermis, less parakeratosis, and less lymphocyte infiltration. PF suppressed IL-6, IL-17A, and IL-22 mRNA. It also stopped HaCat cells from making IL-22, possibly by blocking the MAPK pathway (Yu et al., 2017). PF inhibited astrocytes and microglia from activating chronic constriction-injured rats. It reduced inflammation-promoting cytokines in the spinal cord, such as TNF-α, IL-1β, IL-6, and chemokine (C-X-C motif) ligand (Zhou et al., 2019b). PF also inhibited IL-8 mRNA expression and secretion by lowering ERK1/2 and Akt phosphorylation in human hepatic sinusoidal endothelial cells (Gong et al., 2015). When LPS was added to human oral keratinocytes, PF inhibited the production of pro-inflammatory cytokines such as TNF-α and IL-6. It also suppressed the phosphorylation of NF-κB p65 and IκBα proteins, which hampered NF-κB and p65 from moving into the nucleus (Yu et al., 2019).

### 5.8 Sesquiterpenes

#### 5.8.1 Parthenolide (PAR)

Feverfew [*Tanacetum parthenium* (L.) Sch. Bip.], an Asteraceae medicinal herb, contains PAR. PAR inhibited the inflammatory response in 3T3-CM-cultured macrophages co-cultured with adipose tissue by downregulating IL-6 and MCP-1. PAR reduced adiponectin and resistin dysregulations in macrophage-conditioned medium-cultured adipocytes. In the same study, PL-administered to high-fat diet (HFD)-fed mice, showed an anti-obese effect, connected to anti-inflammatory responses with the regulation of inflammatory cytokines, and the downregulation of NF-κB and MAPKs and inhibited obesity and obesity-induced inflammatory responses via activation of Nrf2/Keap1 signalling pathway (Kim et al., 2019). To understand anti-inflammatory and anti-cancer effects of PAR, researchers used LPS-induced human leukemia monocytic THP-1 cells and human primary monocytes. IL-12p40, IL-6, IL-1β, IL-8, TNF-α, IL-18, and NO were all reduced by PAR in THP-1 cells, with IC50 values ranging from 1.091–2.620 µM TLR4-mediated MAPK and NF-κB signaling contributed to PAR’s anti-inflammatory effects (Li et al., 2015c). Studies focuses that chronic inflammation causes joint destruction and excruciating pain in rheumatoid arthritis. PAR (4 mg/kg) reduced paw inflammation, bone degradation, and pain-like behavior in moderate collagen antibody-induced arthritis (CAIA) BALB/c mice (Williams et al., 2020).

#### 5.8.2 Zerumbone (ZER)

This phytochemical is mainly found in *Zingiber zerumbet* (L.) Roscoe ex Sm. Oral treatment (100, 250, and 500 ppm) in mice repressed NF-κB and HO-1, causing apoptosis and inhibiting colon cancer growth (Kim et al., 2009). A ZER-rich diet (250 and 500 ppm) reduced lung cancer multiplication by reducing growth, inflammation, and NF-κB and HO-1 expression, killing cancer cells in animals (Kim et al., 2009). ZER reduced iNOS and COX-2 in LPS-stimulated RAW 264.7 cells by inducing the HO-1 pathway, which impacted OA dose-dependently. Chien et al. (2016) showed that ZER (1–5 mg/kg) reduced paw edema and pain in a male SD rat OA model (Chien et al., 2016). It also reduces neuroinflammation, β-amyloid deposition, and behavioral deficits in APP/PS1 mice. MAPK signaling pathway inhibition promoted a phenotypic switch from pro-inflammatory to anti-inflammatory in microglia (Li et al., 2020). Using human U937 macrophages generated by LPS, another study found that ZER decreased the up-regulation of pro-inflammatory mediators such as TNF-α, IL-1β, PGE2, the COX-2 protein, and NF-κB (p65), IκBα, and IKKα/β. ZER suppression of inflammatory markers in macrophages required MyD88, demonstrating its potential as a powerful treatment for inflammatory-mediated immunological diseases (Haque et al., 2018).

### 5.9 Diterpenoids

#### 5.9.1 Ginkgolides (GB)

Maidenhair tree extract is a common and old herbal remedy (*Ginkgo biloba* L., Ginkgoaceae) where ginkgo flavonol glycosides (GFGs) and ginkgolides are active ingredients (GGs). GGs include ginkgolide A (GA), ginkgolide B (GB), ginkgolide C (GC), ginkgolide J (GJ), ginkgolide M (GM), ginkgolide K (GK), ginkgolide L (GL), ginkgolide P (GP), ginkgolide Q (GQ), and bilobalide. Hypoxic-ischemic injury to the brain is a significant cause of mortality and severe neurologic disability. One report showed that GB reduced NLRP3 expression in microglia in a rat pup model of hypoxic-ischemic brain injury and stopped Caspase-1 and NF-κB P65 from entering the nucleus. NLRP3 inflammasome activation was less likely (Chen et al., 2018b). Clinical therapy can alleviate myocardial ischemia/reperfusion (MI/R) illnesses by reducing inflammation. Male SD rats with left anterior descending coronary (LAD) artery blockage mimicked MI/R damage. GC may provide an alternative therapy for MI/R disorders by suppressing the CD40-NF-κB signal pathway and downstream inflammatory cytokine production (Zhang et al., 2018a). GB inhibited inflammation and protected LPS-induced chondrocytes by upregulating synthesis-related genes and downregulating matrix-degrading genes to increase chondrocyte collagen II and aggrecan expression and reduced LPS-induced MAPK activation (Hejia et al., 2018).

### 5.10 Triterpenoids

#### 5.10.1 Ursolic acid (UA)

Basil, rosemary, sage, apples and pears may contain this phytochemical in *Glechoma hederacea* L. (Lamiaceae). It was reported that UA decreased TNF-α production in RAW 267.4 macrophages, A549 alveolar epithelial infected with *Mycobacterium tuberculosis* H37Rv, and mouse splenocytes stimulated with Con A. UA activity reduces the levels of COX-2 and NO synthase in stimulated cells. Finally, UA may be future tuberculosis and antibiotic therapy due to its anti-inflammatory properties (Zerin et al., 2016). Inflammation in the brain may play a role in Parkinson’s. The UA therapy reversed neuroinflammation and neurodegeneration and improved biochemical and behavioral indicators. In Parkinson’s mice models, researchers used UA (25 mg/kg) to reduce MPTP-induced neuroinflammation and inflammatory markers (Iba1 and TNF-α) and transcription factor NF-κB (Rai et al., 2019). DSS caused ulcerative colitis in male BALB/c mice, causing colon damage. DSS increased IL-1β and TNF-α, MDA, and SOD in colon homogenate. UA restored DSS’s effects and reduced NF-κB levels in colon tissue (Liu et al., 2016a).

#### 5.10.2 Escin

Horse chestnut extract (*Aesculus hippocastan*um L., Sapindaceae). The glucocorticoid receptor in escin gel may be anti-inflammatory. Both paw edema and capillary permeability rat models treated with escin gel had elevated glucocorticoid receptor levels and reduced NF-κB mRNA (Zhao et al., 2018). Intragastric escin (0.45, 0.9, or 1.8 mg/kg) reduced Indomethacin-induced gastric ulceration in Swiss albino mice, reducing MDA, TNF-α, and VCAM-1. In the same assay, intragastric escin inhibited myeloperoxidase, superoxide dismutase, catalase, and glutathione peroxidase (Wang et al., 2014). In cecal ligation and puncture (CLP) induced intestinal mucosal injury in a mouse model, a low dose of escin ameliorated endotoxin-induced liver injury and intestinal mucosal injury and increased the expression of tight junction protein claudin-5. They add to evidence that escin is a potent anti-inflammatory agent that reduces intestinal mucosa damage in animal models (Li et al., 2015b).

### 5.11 Steroidal compounds

#### 5.11.1 Withaferin A (WA)

WA is a steroidal lactone in Ashwagandha [*Withania somnifera* (L.) Dunal, Solanaceae] with many biological effects. Obesity gives rise to insulin resistance and endothelial dysfunction by the activation of inflammatory pathways. Endothelial cells treated with WA reduced TNF-α and IL-6 production in palmitic acid (PA)-induced insulin-resistant human umbilical vein endothelial cells. When used to treat PA, WA decreased endothelin-1 and plasminogen activator inhibitor type-1 levels and restored endothelium-mediated vasodilation. In the presence of acetylcholine-stress relief (Batumalaie et al., 2016). CNS affects the immune response to infections, traumas, or diseases. WA may treat neuroinflammatory and stress-related diseases. WA reduces astrocyte NF-κB activity and TNF-α, COX-2, and iNOS production in response to LPS/TLR4 pathway activation (Martorana et al., 2015). BALB/c mice given WA (10 mg/kg) improved neurobehavioral function and reduced spinal cord histological changes. WA increased TGF-1β and IL-10 while decreasing IL-1β and TNF-.α (Yan et al., 2017). WA reduced ovalbumin-induced lung damage and fibrosis in mice. WA reduced inflammation-inducing cell infiltration into bronchoalveolar lavage fluid, pro-inflammatory cytokine production, and inflammasome activation via the NLRP3 pathway in human lungs (Zhao et al., 2019). Pulmonary fibrosis is an interstitial lung disease evidenced by chronic inflammation. WA (2 and 4 mg/kg) decreased connective tissue growth factor, collagen 1A2, collagen 3A1, and fibronectin in a bleomycin-induced lung fibrosis mouse model where it reduced NF-κB p65, IL-1β, and TNF-α expression (Bale et al., 2018). People often take too much acetaminophen, which causes liver damage. Our team looked at the hepatoprotective effects of a withanolide-rich fraction (WRF) from *Withania somnifera* (L.) Dunal contains WA (12.9 mg/gm). Male Wistar rats given acetaminophen were given 50, 100, or 200 mg/kg of WRF, which stopped the TNF-α, IL-1β, COX-2, and iNOS proteins from causing inflammation and oxidative stress (Devkar et al., 2016).

#### 5.11.2 β -sitosterol (BSS)

It’s found in wheat germ, rice bran, flax seeds, peanuts, and soybeans (*Glycine max* (L.) Merr., Fabaceae). In murine J774A.1 macrophage, BSS reduced pro-inflammatory cytokines and chemokines and increased anti-inflammatory IL-10. NF-κB translocation to the nucleus was inhibited by protein tyrosine phosphatase (SHP-1) (Valerio and Awad, 2011). BSS nanoparticles (7.5–30 µM) prevented keratinocytes and macrophages from releasing TNF-α, IL-1β, IL-6, IL-8, and ROS when triggered by peptidoglycan, TNF-α, or LPS. Also, BSS decreased NLRP3, a key part of NLRP3 inflammasomes, and stopped caspase-1 (Liao et al., 2018). In CIA mice, intraperitoneal BSS (20 or 50 mg/kg) or adoptive transfer of BSS-BMDMs reduced ankle swelling, collagen-specific antibodies (IgG and IgG1), and pro-inflammatory cytokines (Liu et al., 2019a).

### 5.12 Curcuminoids

#### 5.12.1 Curcumin

Turmeric’s roots contain curcumin (*Curcuma longa* L., Zingiberaceae) which adds flavor to food and has medical uses. Curcumin protects neurons and slows microglia and macrophage activation and death. In male C57BL/6 mice with traumatic brain injury, TLR4/MyD88/NF-κB signaling was involved (Zhu et al., 2014). Curcumin’s effects on myocarditis were studied in rodents where it inhibited phosphoinositide 3-kinase (PI3K)/Akt/NF-κB signaling in coxsackievirus B3-induced myocarditis mice. It also inhibited inflammatory cytokines like TNF-α, IL-6, and IL-1β, reducing inflammation (Song et al., 2013). Neuroinflammation contributes to AD. Curcumin’s anti-inflammatory effects may aid AD patients. Liu et al. (2016b) found curcumin improved mice’s spatial memory and cholinergic neurons. This improvement was related to NF-κB signaling pathways and PPARγ mediated transcription (Liu et al., 2016b). Curcumin and curcumol were also tested on macrophage cells exposed to cigarette smoke extract. It was found that curcumol and curcumin inhibited the NF-κB signaling pathway and downregulated proinflammatory factors (Li et al., 2019). BALB/c mice given ovalbumin developed asthma. Curcumin (20 mg/kg and 100 mg/kg) reduced inflammatory cell infiltration, goblet cell hyperplasia, alveolar thickening, edema, and vascular congestion in BALB/c with ovalbumin-induced allergic asthma; and decreased mRNA expression levels of cytokines IL-4, IL-5, TNF-α, TGF-β (Shahid et al., 2019). Lipoteichoic acid (LTA) stimulates neuroinflammatory molecules, contributing to neurodegeneration. In LTA-stimulated BV-2 microglial cells, curcumin’s anti-inflammatory effects decreased TNF-α, PGE2, NO, iNOS, and COX-2. Another study found that curcumin reduced LTA-induced phosphorylation of MAPK, ERK, p38, Akt and NF-κB translocation. Curcumin stimulated HO-1 and Nrf-2 expression in microglial cells (Yu et al., 2018).

### 5.13 Stilbenes

#### 5.13.1 Resveratrol (RSV)

Red grapes (*Vitis vinifera* L., Vitaceae) and wine have one of the anti-inflammatory polyphenols known as resveratrol (RSV). A review concluded the multifaceted approach of RSV such as activation of protein-1 (AP-1), NF-κB, Cox-2 and regulation of proinflammatory cytokines like IL-6, IL-8, IL-10 and TNF-α as well as ICAM-1 and MCP expression (Latruffe et al., 2015). RSV inhibited ICAM-1, iNOS, and IL-1β mRNA expression in TNF-α-treated human coronary endothelial cells, demonstrating anti-inflammatory properties (Huang et al., 2017). RSV also improved lung histological damage and decreased pro-inflammatory cytokines (IL-6, IL-17, TNF-α, and TGF-β) in cigarette smoke chronic obstructive pulmonary disease (COPD) animals (Chen et al., 2016). RSV improves circulation in streptozotocin-treated rats, a pancreatic cell toxin. The improvement was associated with lower blood levels of TNF-α, IL-1β, and IL-6 and suppression of vascular endothelial growth factor (VEGF) via the p38-MAPK and NF-κB pathways (Yan et al., 2018). Yanez et al. (2019) examined the effects of RSV and nicotinamide on the downregulation of high levels of TNF-α, IL-6, and VEGF in LPS-induced macrophages. Nicotinamide increased RSV-induced PARP1 activation and its related anti-inflammatory effects, which were mediated through B-cell lymphoma 6 upregulation and COX-2 downregulation (Yanez et al., 2019).

### 5.14 Phenolic acids

#### 5.14.1 Rosmarinic acid (RosA)

RosA is an ester of caffeic acid and 3, 4-dihydroxyphenyl lactic acid found in rosemary herb (*Rosmarinus officinalis* L., Lamiaceae). Rahbardar et al. (2017) found that RosA (40 mg/kg) decreased spinal inflammatory markers, including matrix MMP-2, PGE-2, IL-1β, and COX-2, in rats with sciatic nerve CCI-induced neuropathic pain (Rahbardar et al., 2017). Cao et al. (2016) reported that RosA (75, 150, and 300 mg/kg) reduced TNF-α, IL-6, IL-1β, TGF-β, and VEGF in HCC while NF-κB and p65 was also decreased in the xenograft microenvironment (Cao et al., 2016). RosA from pomegranate peel reduced TNF-α in Freund’s complete adjuvant-induced arthritis by increasing GSH and SOD while reducing MDA levels (Gautam et al., 2019). Jin et al. (2017) found that RosA reduced DSS-induced colon shortening and splenomegaly in mice. RosA prevented COX-2 and iNOS expression and IL-1β, IL-6, and IL-22 production in inflamed mucosa by inhibiting NF-κB, p65, and pSTAT3 expression and nuclear transport (Jin et al., 2017). One of the research also described RosA anti-inflammatory effect on LPS-induced mouse mastitis and mouse mammary epithelial cells. It reduced myeloperoxidase activity, TNF-α, IL-1β, and IL-6 levels (Jiang et al., 2018).

#### 5.14.2 Ellagic acid (EA)

Ellagic acid (EA) is present in fruits, such as pomegranates (*Punica granatum*, Lythraceae), seeds, and vegetables. Innate immunity plays an important role in managing oral cavity homeostasis, infections, and cancers. Promsong et al. (2015) measured the effects of EA (12.5–100 μM) on innate immune mediators in primary human gingival epithelial cells (HGEs). EA increased the expression of RANTES (regulated on activation of normal T-cell expressed and secreted), IL-1β, and IL-2, while decreased TNF-α, C-C Motif Chemokine Ligand 20 (CCL20), IL-6, IL-8, and C-X-C Motif Chemokine Ligand 5 (CXCL5) (Promsong et al., 2015). In a different study, EA (50, 100, and 150 mg/kg) decreased the levels of blood glucose, TNF-α in serum, and the expression levels of TLR-4, IL-1 receptor-associated kinase 4 (IRAK4), TNF-receptor associated factor 6 (TRAF-6), IKK-β-, and NF-κB p65 in the kidney tissue of mice with streptozotocin-induced diabetic nephropathy (Zhou et al., 2019a). Guan et al. (2017) studied EA’s effects on LPS-induced lung damage in mice. He found that EA (5 mg/kg) reduced LPS-induced protein dispersion in bronchoalveolar lavage fluid and inflammatory cell infiltration into lung tissue while reduced TNF-α, IL-6, and IL-1β and increased IL-10 (Guan et al., 2017). One more research evidenced that treatment with EA (50 mg/kg) reduced paw swelling, inflammation, NF-κB, IL-1β, MMP-9, VEGF, caspase-3 expression, blood oxidative stress, and NO levels in a rat model of adjuvant-induced arthritis (Fikry et al., 2019). In addition, pomegranate peel extract high in EA inhibited the generation of IL-17 by activated T cells isolated from mice with experimental autoimmune encephalomyelitis (Stojanović et al., 2017). Furthermore, wistar rat hippocampi were exposed to arsenic, which caused neuroinflammation and mitochondrial dysfunction. EA reduced arsenic-induced neurotoxicity in rats by reducing ROS, Bax, Bcl2, and inflammatory biomarkers (IL-1β, TNF-α, IFN-γ) (Firdaus et al., 2018).

#### 5.14.3 Gallic acid (GA)

Gallic acid (GA) is abundant in tea leaves (*Camellia sinensis* (L.) Kuntze, Theaceae), along with gall nuts, apple peels, sumac, green tea, and grapes. Recent research examined the effects of GA on IL-1-induced human intestinal epithelial cell line and 2,4,6-trinitrobenzene sulfonic acid (TNBS)-induced ulcerative colitis (UC) in mice. GA raised the expressions of IL-4 and IL-10, whereas blocking the NF-κB pathway decreased the expressions of IL-1, IL-6, IL-12, IL-17, IL-23, TGF-β, and TNF-α. These modifications alleviated inflammation, reversed the loss in body weight and the rise in colon weight, and mitigated the histological alterations caused by UC (Zhu et al., 2019). Generally, hypertrophic scars are the result of prolonged intense inflammation. Fan et al. (2018) studied GA’s effect on LPS-induced inflammation in hypertrophic scar fibroblasts and reported reduced TNF-α, IL-6, IL-1β, and IL-8 levels. This indicated an inflammatory response via TLR-4/NF-κB/PPARγ pathway (Fan et al., 2018). Endometriosis is a gynecologic disease in women that can cause infertility and chronic pelvic pain with a relatively high recurrence rate. GA (102.4 μg/ml) and its derivatives showed ameliorating effects on endometriosis primary cultures by regulating NF-κB mRNA expression and IL-6 secretions (Bustami et al., 2018).

#### 5.14.4 Protocatechuic acid (PCA)

Protocatechuic acid (PCA) is a phenolic chemical extracted from onion (*Allium cepa* L., Amaryllidaceae) and found in many plants and fruits. Recent research shows PCA’s anti-inflammatory mechanism via sirtuin1(SIRT1)/NF-κB in LPS-activated BV2 microglia (Kaewmool et al., 2020). Inflamed visceral adipose tissue (VAT) causes insulin resistance and T2DM in obese patients. By increasing insulin receptor substrate-1 and Akt phosphorylation, PCA can modulate insulin sensitivity and inflammation in obese-VAT and normal-weight T2DM patients. This may be due to reduced protein tyrosine phosphatase 1B activity in obese-VAT treated with PCA. Thus, PCA is a powerful phytochemical against obesity-related inflammation and IR (Ormazabal et al., 2018). The polarization of macrophages affects atherosclerosis. PCA blocked PI3K-Akt-mediated NF-κB activation and M1 polarization. In J774 cells and mouse bone marrow macrophages, it phosphorylated STAT-6 and activated PPAR-γ, increasing M2 activation. These findings showed PCA relieved atherosclerosis by modulating M1-M2 conversion (Liu et al., 2019b). Benign prostatic hyperplasia (BPH) causes an enlarged prostate. Akanni et al. (2020) reported that BPH castrated rats treated with PA showed reduction in inflammation and oxidative stress and caused histological changes (Akanni et al., 2020).

#### 5.14.5 Vanillic acid (VA)

Vanillic acid (VA) is the major component of the extracts of the vanilla (*Vanilla planifolia* Jacks. ex Andrews, Orchidaceae) bean and pod, commonly utilized in food flavoring agents, cosmetics and drugs. In a mouse model of inflammation produced by carrageenan, VA reduced hyperalgesia, leukocyte recruitment, oxidative stress, IL-33, TNF-α, and IL-1β production, as well as NF-κB activation. This study proves analgesic and anti-inflammatory actions of VA, associated with Nrf2 activation (Calixto-Campos et al., 2015). In another study, VA reduced Aβ1-42-induced oxidative stress, neuroinflammation, and cognitive impairment in mice by activating Nrf2 and increasing HO-1 expression (Amin et al., 2017). The anti-inflammatory potential of VA was evaluated in LPS-induced macrophages and in *in vivo* animal models. VA reduced LPS-induced gene expression and pro-inflammatory mediators, including iNOS/COX-2 and cytokines. The mechanism involved was suppression of NF-κB activation in macrophages and improve acetic acid-induced vascular permeability and zymosan-induced leukocyte migration in mice (Lee et al., 2018).

#### 5.14.6 6-Gingerol (6-G)

This phytochemical is found in ginger (*Zingiber officinale*, Rosc., Zingiberaceae), spice and herbal medicine. 6-G (6 mg/kg) pre-treatment alleviated MI/R in SD rats by improving the cardiac functions. The later involved reduced myocardial infarction area and cardiac pathological injury, lowered myocardial enzyme level and inhibited inflammatory response by upregulating PI3K and p-Akt expression (Xu et al., 2018b). Additionally, 6-G rich fraction inhibited the inflammatory markers such as myeloperoxidase, NO, and TNF-α in brains, ovaries, and uterus of chlorpyrifos-treated rats (Abolaji et al., 2017). A report assessed 6-G inhibition on IL-1 induced osteoclast differentiation in co-cultures of osteoblasts and osteoclast precursor cells and found that 6-G suppressed NF-κB ligand and reduced PGE2, indicating its potential use in the treatment of inflammatory bone destruction associated with excessive PGE2 production (Hwang et al., 2018). The AD model of whiskers rats produced by streptozotocin was investigated to examine whether 6-G therapy might reduce inflammation and ameliorate cognitive impairment. The researchers observed that pre-treatment with 10 and 20 mg/kg 6-G decreased levels of neuroinflammatory and α, β-secretases, APH1a (Aph-1 Homolog A, Gamma-Secretase Subunit), and COX-2, resulting in an improvement in cognitive behaviors (Halawany et al., 2017). 6-G (25 mg/kg) antioxidant and anti-inflammatory properties protected rat kidneys from septic acute damage by reducing ROS, RNS, MDA and increasing GSH activity (Rodrigues et al., 2018). Additionally, orally administered 6-G rich extract reduced the levels of the proinflammatory marker TNF-α and expression of NF-κB and vascular endothelial growth factor in the retinal tissue of the streptozotocin-induced diabetic Wistar albino rats (Dongare et al., 2016).

#### 5.14.7 Caffeic acid phenethyl ester (CAPE)

It’s a polyphenolic chemical mostly found in black poplar (*Populus nigra* L., Salicaceae) and beehive propolis. Glaucoma is characterized by the death of retinal ganglion cells (RGCs) and is a leading cause of blindness worldwide. Jia et al. (2019) reported that CAPE inhibits NF-κB activation, reduces the production of inflammatory cytokines like IL-8, IL-6, iNOS, COX-2, TNF-α, and C-C ligand-2 in a glaucoma rat model of optic nerve crush (Jia et al., 2019). One more study found that CAPE inhibits NF-κB activation via thiol group modification and p65 phosphorylation in RAW 264.7 cells (Takakura et al., 2018). In the host’s defense against dental caries, odontoblasts produce growth factors and develop reparative dentin. CAPE increased VEGF mRNA expression and production in rat odontoblast-like KN-3 cells and enhanced NF-κB transcription factor. Thus, CAPE is predicted as a unique biological material for dental pulp treatment (Kuramoto et al., 2019). Salles et al. (2019) showed that treatment with CAPE (10 µM) improved wound inflammatory and oxidative profile with decreased TNF-α, phosphorylated NF-kB p65 protein, NOS2 and COX-2 expression in male Swiss diabetic rats (Salles et al., 2019). Periodontal disease is linked to chronic oxidative stress and inflammation. It was reported that in primary murine macrophages, CAPE showed antioxidative effects via the Nrf2-mediated HO-1 pathway and anti-inflammatory effects via NF-κB suppression (Stähli et al., 2019).

## 6 Phytochemicals evaluated in clinical trials

**FIGURE 4:**
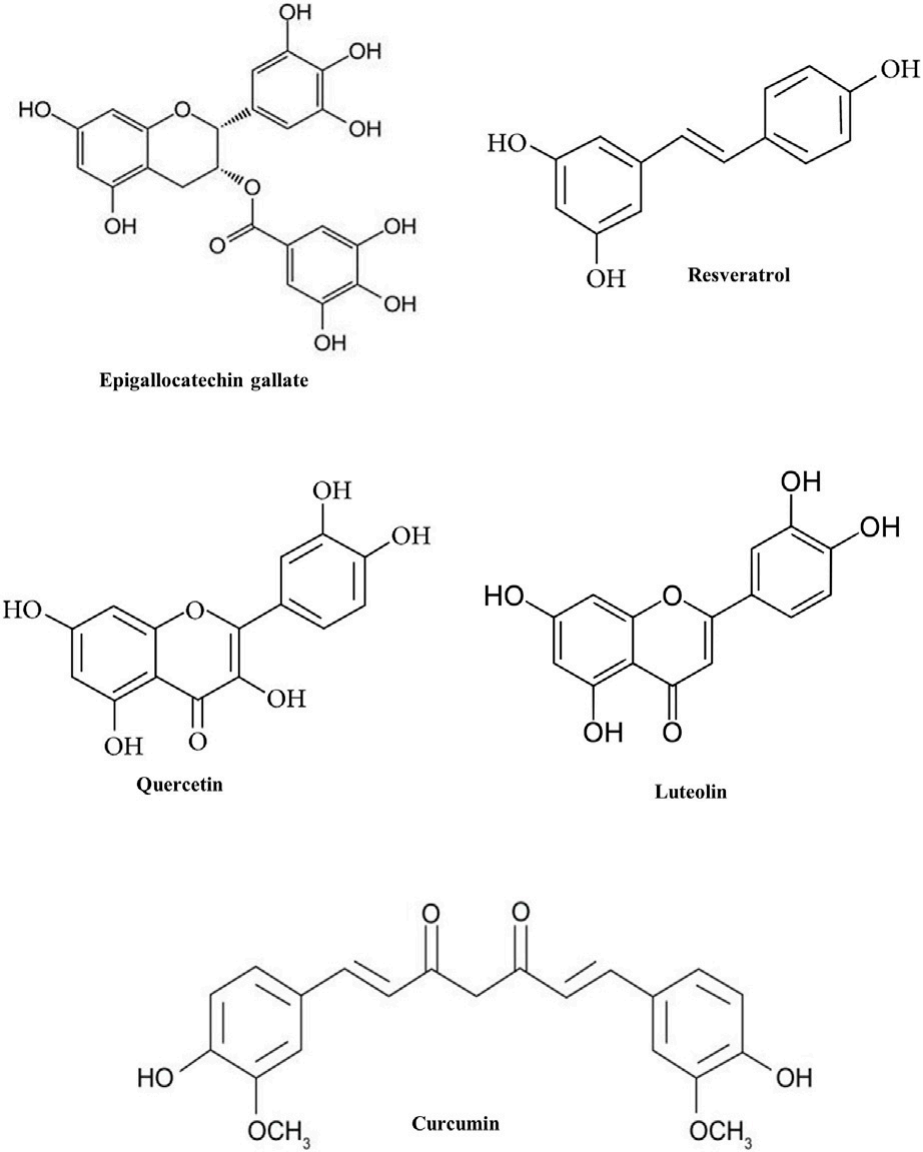
Chemical structure of some phytochemicals used in clinical trials.

The effectiveness of phytoconstituents for different health complications is known since ancient days. Recent advances in research has provided a larger platform to find out the efficacy and mechanism of these plant-derived components. Animal models are, of course, invaluable to study the pharmacological capacity of a drug. However, these models do not satisfactorily represent the human conditions and have limitations. In this context, we have summarized some major phytochemicals that are being studied for their role in inflammation in different complications and are undergoing clinical trials as well (Figure 4; Table 4).

**TABLE 4:** List of anti-inflammatory phytochemicals used in clinical trials.

Phytochemicals	Class of compound	Disease/disorder	Assessment	References
Resveratrol	*Vitis vinifera* (Vitaceae)	Gulf War Illness	Improvements in cognitive functioning, functional status, mood, hippocampal neurogenesis, and functional connectivity as well as anti-inflammatory and antioxidant effects	NCT03665740^a^
Curcumin	*Curcuma longa*, (Zingiberaceae)	Bladder Spasm	Reducing inflammation for ureteral stent-induced symptoms	NCT02598726^a^
		Malignant Neoplasm		
		Pain		
		Urinary Urgency		
Epigallocatechin gallate	Camellia sinensis (Theaceae)	Obesity	To assess endotoxin and inflammatory biomarkers	NCT03413735^a^
		Endotoxemia		
		Inflammation		
Quercetin	Malus domestica, (Rosaceae)	COVID-19	Prophylaxis and treatment of COVID-19	NCT04377789^a^
Luteolin	Rosmarinus officinalis (Lamiaceae)	Frontotemporal Dementia	To assess the brain correlates related to the clinical improvement associated with PEA-LUT treatment	NCT04489017^a^

^a^ Indicates reference found at www.clinicaltrials.gov with corresponding identifier code (NCT).

### 6.1 Resveratrol

Resveratrol clinical research focuses on T2DM/metabolic syndrome, polycystic ovary syndrome, and non-alcoholic fatty liver disease. This phytochemical activates SIRT1 and may help with metabolic, inflammatory, and cell cycle disorders. A low grade of systemic inflammation and oxidative damage can be seen in smokers. Bo et al. (2013) reported that resveratrol (500 mg/day) reduced CRP and TGs and improved antioxidant status in 50 healthy adult smokers during a 90-day cross-over, randomized and double-blind study. These effects were depending on anti-inflammatory and anti-oxidant properties of resveratrol that ultimately subsided cardiovascular risk in participants (Bo et al., 2013). In another study, healthy Japanese participants were given resveratrol (1 g/day for 28 days) to examine its effects on immune cells. Here, increased γδ-T cells and regulatory T cells reduced plasma TNF-α and MCP-1 levels (Espinoza et al., 2017). In a 24-week randomized controlled trial, 93 veterans participated to evaluate cognitive functioning, functional status, mood, hippocampal neurogenesis, and functional connectivity, as well as the anti-inflammatory and antioxidant effects of resveratrol (500 mg, 1,000 mg, 1,500 mg, and 2,000 mg) (NCT03665740). Study results have not been posted yet.

### 6.2 Curcumin

Curcumin, also known as diferuloylmethane is turmeric’s main component (*Curcuma longa* L., Zingiberaceae) and used to treat inflammatory illnesses in Ayurvedic medicine. A randomized controlled trial evaluated curcumin’s anti-inflammatory effects in people with metabolic syndrome. Here, 117 participants received curcumin (1 g/day) or a placebo for 8 weeks and showed reduction in TNF-α, IL-6, TGF-β, and MCP-1 in blood (Panahi et al., 2016). A report found that peptic ulcers treated with 600 mg of curcumin per day for 12 weeks improved the condition from 48% to 76%, depending on treatment length (Prucksunand et al., 2001). The function of inflammation in the development of pancreatitis and subsequent tissue damage is crucial (Vaquero et al., 2001). A 6-week pilot study of tropical pancreatitis with 15 patients was performed where administration of curcumin (5 mg/day) with piperine (5 mg) reduced MDA levels, but there was no significant differences in GSH or pain scores as compared to placebo group (Durgaprasad et al., 2005). In another study, forty cancer patients are being examined in a phase I pilot study to examine the adverse effects and optimal dose of curcumin when combined with piperine extract to reduce ureteral stent-induced symptoms (NCT02598726).

### 6.3 Epigallocatechin gallate (EGCG)

EGCG, also known as epigallocatechin-3-gallate, is a component of green tea, *Camellia sinensis* (L.) Kuntze (Theaceae). The inflammatory nature of MS increases IL-6 levels in the blood that elevates and often exacerbates pain associated with a physical disability. In a pilot trial, the effects of coconut oil and EGCG on IL-6, anxiety, and functional impairment in MS patients were evaluated. 51 patients with MS were given EGCG (800 mg) and coconut oil (60 ml) for 4 weeks following the Mediterranean diet. The results showed improvement in anxiety and functional capacity along with a decrease in IL-6 (Platero et al., 2020). One more study uses catechin-rich green tea and is being tested on 40 humans to improve gut barrier function and prevent endotoxin translocation and inflammation (NCT03413735).

### 6.4 Quercetin

Quercetin, a flavonol and plant secondary metabolite found in apples, grapevines, berries, broccoli, onions, and capers. Quercetin targets prominent pro-inflammatory signaling pathways such as STAT1, NF-κB, MAPK and scavenges reactive oxygen and nitrogen species (Hämäläinen et al., 2007). It has been postulated that oxidative stress and low antioxidant levels cause inflammatory sarcoidosis. It was reported that quercetin (15 mg per day) treatment reduced inflammation and boosts antioxidant defense by increasing total plasma antioxidant capacity in sarcoidosis patients participated in double-blind study (Boots et al., 2011). In a randomized, double-blind 8 weeks study, subjects with systematic and regular exercise showed reduction in oxidative stress and inflammatory markers CRP and IL-6 upon treatment with quercetin alone (500 mg) and/or with vitamin C (250 mg) (Askari et al., 2012). COPD is a chronic pulmonary condition that affects millions of people worldwide and reduction of oxidative stress and inflammation are essential part of COPD management (King, 2015). Quercetin (2000 mg/day) efficacy is being evaluated using IL-β, IL-8, bronchoalveolar lavage, CRP, and surfactant protein-D involving 15 COPD patients in a double-blind, placebo-controlled study (NCT03989271). The coronavirus emerged in late 2019, caused multiple deaths via a disease called COVID-19 with challenging health burden around the globe (Zhou et al., 2020). Based on quercetin’s strong scavenger and anti-inflammatory activity, some researchers hypothesized it could prevent and treat COVID-19. The randomized clinical trial included 50 participants with COVID-19 infection and a 1,000 mg/day quercetin dose (NCT04377789).

### 6.5 Luteolin

Luteolin is a flavone found in carrots, cabbage, artichoke, tea, and celery while it is used majorly for cancer and inflammation due to its antitumor and anti-inflammatory properties. A correlation has been found between autism spectrum disorders (ASD) and cognitive function-related brain inflammation (Pardo et al., 2005; El-Ansary and Al-Ayadhi, 2012). Taliou et al. (2013) reported that treatment with luteolin (100 mg/10 kg) effectively reduced ASD symptoms in children in a 6 week pilot research using an open-label design (Taliou et al., 2013). Frontotemporal dementia (FTD) is a disease where neuroinflammation may play a role and that neuroinflammation-targeting medications may be effective in treating this condition (Cordaro et al., 2020). A clinical trial is being conducted with 50 FTD patients to evaluate palmitoylethanolamide mixed with luteolin (PEA-LUT) at 700 mg × 2/day for 24 weeks (NCT04489017).

## 7 Phytochemicals used currently in inflammatory diseases/disorders

**FIGURE 5:**
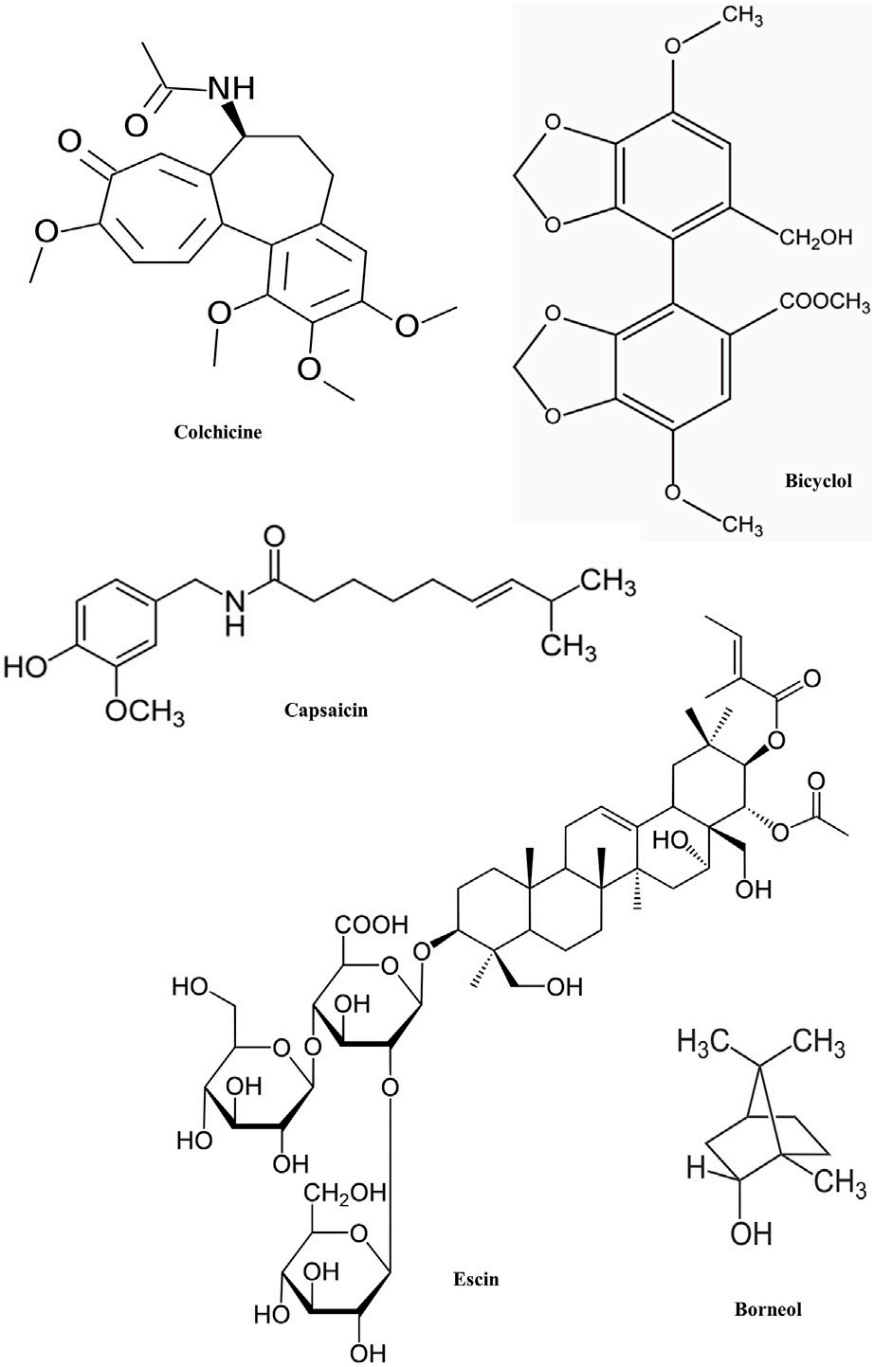
Chemical structure of phytochemicals used as current drugs/medicine.

Natural products are a vital resource for global pharmaceutical firms developing new medicines. About 25% of this natural resource comes from pharmaceuticals i) A direct supply of therapeutic substances (both pure medications and phytomedicines); ii) raw materials for manufacturing complex, semi-synthetic therapeutics; iii) models for developing lead compounds; and iv) taxonomic markers for discovering novel drugs (Calixto, 2019). In *in vitro* and *in vivo* studies, many phytochemicals have shown anti-inflammatory activity, and most have been tested in clinical trials. Not all are approved as medicines/drugs; but are used as supplements. Using phytochemicals as drugs or medicines depends on country norms. In this review, we list some effective anti-inflammatory drugs used around the world (Figure 5; Table 5).

**TABLE 5:** List of anti-inflammatory phytochemicals used as current drugs/medicines.

Drug/medicine (class/group of compounds)	Pharmacological action	Disease/disorder	Molecular targets	References
Colchicine (alkaloid)	Microtubule polymerization by binding to tubulin	Gout attacks Joint Pain	Tubulin	Leung et al. (2015)
Escin (triterpenoid saponin)	Anti-inflammatory, reduces vascular permeability by inducing endothelial nitric oxide synthesis	Cerebral edema and chronic venous insufficiency	NO synthesis	Gallelli (2019)
Capsaicin (methoxy phenol)	Defunctionalisation of nociceptor fibers by inducing a topical hypersensitivity reaction on the skin	Neuropathic pain associated with postherpetic neuralgia	Nociceptor fibers	Fattori et al. (2016)
Bicyclol (lignan)	cytochrome P-450 enzyme system stimulants	Liver complications	cytochrome P-450 enzyme	Liu et al. (2005); Bao and Liu (2008)
Borneol (monoterpene)	Induces anesthesia and analgesia	Anxiety, fatigue, and insomnia	—	Xiong et al. (2013); Ji et al. (2020)
Bromelain	Reduces inflammation by interfering with the enzymatic synthesis involved in the arachidonic acid metabolic pathway	Osteoarthritis, hay fever, ulcerative colitis, and debridement	Arachidonic acid	Hale et al. (2005); Rathnavelu et al. (2016)

### 7.1 Colchicine

Colchicine is an alkaloid of *Colchicum autumnale* L. (Colchicaceae), also called autumn crocus or meadow saffron. This phytochemical is an alternative medication for those who are unable to tolerate NSAIDs in gout. Colchicine prevents microtubule polymerization by binding to tubulin and suppressing leukocyte and other inflammatory cell proliferation and reduces urate crystal inflammation (Leung et al., 2015).

### 7.2 Escin

Escin is a horse chestnut triterpenoid saponin (*Aesculus hippocastanum* L.), which is known for its vasoprotective, anti-inflammatory, anti-edematous, and anti-nociceptive properties. Traditional Chinese medicine uses escin to treat cerebral edema and chronic venous insufficiency. Recent research shows that escin can reduce vascular permeability in inflamed tissues, preventing swelling (Gallelli, 2019).

### 7.3 Capsaicin

Various non-steroidal drugs and phytochemicals are analgesics and anti-inflammatory agents (Kim et al., 2003). Capsaicin is a topical analgesic approved by the FDA for alleviating the neuropathic pain associated with postherpetic neuralgia. It's available in cream, powder, and patch forms, but also present in some nutritional supplements. The exact mechanism of action is not known, however it is attributed to the defunctionalisation of nociceptor fibres by inducing a topical hypersensitivity reaction on the skin (Fattori et al., 2016).

### 7.4 Bicyclol

A synthetic compound derived from Schisandra C, a lignan extracted from the Chinese medicinal herb *Schisandra chinensis* Fructus (Turcz.) Baill. Chinese Medical Association approved this anti-inflammatory drug for liver complications. Mechanisms of action include cytochrome P-450 stimulants, free radical-scavenging HSP70 stimulants, and protein kinase C inhibitors (Liu et al., 2005; Bao and Liu, 2008).

### 7.5 Borneol

Borneol is present in many essential oils and it’s a bicyclic monoterpene with a strong, bitter aroma and flavor. Research shows borneol’s effectiveness in inflammation and related complications (Ji et al., 2020). For instance, in Chinese medicine, borneol treats anxiety, fatigue, and insomnia. Borneol not only causes anesthesia, pain relief for abdominal pain, wounds, and burns but also treats rheumatism, hemorrhoids, skin diseases, and ulcers. More precisely, it is well known to relieves pain, inflammation, digestive issues, stress, and anxiety (Xiong et al., 2013).

### 7.6 Bromelain

Bromelain is a group of protein-digesting enzymes found in pineapple juice and the pineapple stem. In the US, it's a dietary supplement, but elsewhere it is a medicine. Bromelain stimulates inflammatory pathways to produce pain and inflammation-fighting substances. Hence it is generally prescribed for osteoarthritis, hay fever, ulcerative colitis, and debridement (Rathnavelu et al., 2016). It stops the release of IL-1β, IL-6, and TNF-α by activated immune cells when inflammation causes them to make too many cytokines (Hale et al., 2005).

## 8 Discussion and conclusion

This review briefly describes recent investigations on the anti-inflammatory properties of medicinal phytochemicals using preclinical and clinical studies. The preclinical studies of these phytochemicals have led to a better understanding of their mode of action for the therapeutic management of a variety of chronic inflammatory diseases and disorders and steered the way to the development of many anti-inflammatory drugs which are being used clinically. It is evidenced that phytochemicals may suppress the expression of proinflammatory genes and stimulate the expression of anti-inflammatory genes; this differential gene expression is governed by epigenetic changes. In this study, we demonstrate that phytochemicals exert their anti-inflammatory impact by modulating the expression of proinflammatory miRNAs, particularly those that are increased after NF-κB activation. These phytochemicals also modulate key inflammatory signaling pathways, such as MAPKs, STAT, and Nrf-2.

Additionally, the present review gave insights towards the relation of inflammation and obesity, with one causing the other. Some of the studies suggested in preclinical studies gives evidence of the linkage between inflammation and obesity. For example, obesity mice model treated with apigenin showed reduction in body weight along with improvement in inflammatory parameters (Gentile et al., 2018). One more study proved that PL administration showed anti-obese effect and inhibited obesity-induced inflammatory responses (Kim et al., 2019). We have also discussed how inflammatory conditions are linked with birth complications that decide future disease/disorders in neonatal stage. In fact, during pregnancy, mother provides a variety of food and conventional nutrients that contain a variety of phytochemicals in various concentrations to the foetus. It also indicates that concentration-dependent effects of phytochemicals must be present to control the repercussions of mother’s health and food habits.

It is of utmost interest to understand the specific role of phytochemicals in different inflammatory diseases rather than depending upon the crude extracts or partially purified mixture of phytochemicals. It is also important to understand the right time for the intervention by phytochemicals in different diseases. It’s very likely that the same phytochemicals may not be effective at different ages for a similar inflammatory disorder. The clinical studies are not addressing in detail the above facts regarding phytochemicals intervention, specifying the needs for controlled treatment with conventional allopathic drugs. This kind of study may trigger the competitive use of phytochemicals against allopathic drugs also. Finally, it is important to discuss and study the above fundamentals to better understand the mechanism of action of phytochemicals in inflammation associated diseases and disorders. Another treatment modality is combination therapy that combines two or more therapeutic agents such as certain specific phytochemicals with known therapeutic effects. Combination therapy is the cornerstone of cancer treatment where a combination of anticancer drugs is used to enhance treatment efficacy compared to the monotherapy because a combination has the potential to target key signaling pathways that control tumor growth where synthetic drugs are used with one or mixture of the phytochemicals. The application of complementary and alternative medicine, which includes phytochemicals and herbal extracts that leads to chances of herb-drug interactions (HemaIswarya and Doble, 2007). In another study, anticancer activities of each of the three phytochemicals baicalein, curcumin, and resveratrol in combination with a chemotherapy drug paclitaxel indicated that combination of paclitaxel with curcumin showed synergistic growth inhibition and significant apoptosis in human breast cancer MCF-7 cell lines (Zhan et al., 2014).

The use of phytochemicals as therapeutic agents has certain limitations that deserve some attention, such as the larger dose requirement for some compounds, poor solubility, isolation, and procurement, etc. In fact, most of the clinical trials do not take into consideration the inflammatory parameters in the assessment. A small number of phytochemicals which have been approved for clinical trials, are essentially those that have already been tested in preclinical studies as anti-inflammatory molecules. On the contrary, a large number of phytochemicals are being used as supplements and are available over the counter, are also found to be effective but these are not approved as medicines/drug due to their lack of proper clinical evaluation. Finally, it seems that there is a broad difference in basic preclinical studies of these anti-inflammatory phytochemicals and their availability as drugs/medicines. For future perspectives, it looks like that the design of the study should be more specific at the molecular level and more clinical trials should be introduced through targeted treatments with therapeutic phytochemicals. Furthermore, *in silico* studies can be initiated to increase the spectrum of the study as well as to find more pronounced details regarding the feasibility and therapeutic usefulness of these phytochemicals. Finally, this review has focused on those phytochemicals which are at the preclinical and clinical level and summarized the mechanism of action of these phytochemicals at the molecular level. It is expected that present study will provide the necessary understanding to define specific phytochemicals with anti-inflammatory properties that can be used as therapeutics in complex diseases such as obesity, diabetes, and cancer.
